# A Novel Zinc Exporter CtpG Enhances Resistance to Zinc Toxicity and Survival in Mycobacterium bovis

**DOI:** 10.1128/spectrum.01456-21

**Published:** 2022-04-04

**Authors:** Liu Chen, Xiaohui Li, Piao Xu, Zheng-Guo He

**Affiliations:** a College of Life Science and Technology, Huazhong Agricultural University, Wuhan, China; b State Key Laboratory for Conservation and Utilization of Subtropical Agro-Bioresources, College of Life Science and Technology, Guangxi University, Nanning, China; Johns Hopkins University School of Medicine

**Keywords:** *Mycobacterium bovis*, CtpG, zinc toxicity, intracellular survival, P1B-type ATPase

## Abstract

Zinc is a microelement essential for the growth of almost all organisms, but it is toxic at high concentrations and represents an antimicrobial strategy for macrophages. Mycobacterium tuberculosis and Mycobacterium bovis are two well-known intracellular pathogens with strong environmental adaptability, including zinc toxicity. However, the signaling pathway and molecular mechanisms on sensing and resistance to zinc toxicity remains unclear in mycobacteria. Here, we first report that P_1B_-type ATPase CtpG acts as a zinc efflux transporter and characterize a novel CmtR-CtpG-Zn^2+^ regulatory pathway that enhances mycobacterial resistance to zinc toxicity. We found that zinc upregulates *ctp*G expression via transcription factor CmtR and stimulates the ATPase activity of CtpG. The APC residues in TM6 is essential for CtpG to export zinc and enhance M. bovis BCG resistance to zinc toxicity. During infection, CtpG inhibits zinc accumulation in the mycobacteria, and aids bacterial survival in THP-1 macrophage and mice with elevated inflammatory responses. Our findings revealed the existence of a novel regulatory pathway on mycobacteria responding to and adapting to host-mediated zinc toxicity.

**IMPORTANCE** Tuberculosis is caused by the bacillus Mycobacterium tuberculosis and is one of the major sources of mortality. M. tuberculosis has developed unique mechanisms to adapt to host environments, including zinc deficiency and toxicity, during infection. However, the molecular mechanism by which mycobacteria promote detoxification of zinc, and the associated signaling pathways remains largely unclear. In this study, we first report that P_1B_-type ATPase CtpG acts as a zinc efflux transporter and characterize a novel CmtR-CtpG-Zn^2+^ regulatory pathway that enhances mycobacterial resistance to zinc toxicity in M. bovis. Our findings reveal the existence of a novel excess zinc-triggered signaling circuit, provide new insights into mycobacterial adaptation to the host environment during infection, and might be useful targets for the treatment of tuberculosis.

## INTRODUCTION

Tuberculosis (TB) is caused by the bacillus Mycobacterium tuberculosis (Mtb) and is a leading cause of death and disability worldwide. Vaccination with the Bacillus Calmette-Guerin (BCG) and chemotherapy are the common strategies for TB control (1). Mtb is a facultative intracellular pathogen able to survive in an infected host for decades, with little or no replication (2). Thus, the pathogen must exist various strategies to standwith diverse hostile environment within the host, including a toxic environment and nutritional immunity by depriving bacteria from some metals (Fe, Mn) or poisoning the bacteria with others (Cu, Zn) (3–6).

Metals are essential components for nearly all forms of life. Indeed, approximately one-half of all enzymes in organism require a metal cofactor to fulfill their biological activity (7). However, it is toxic at high concentrations and represents a cell-autonomous immunity strategy (3, 8–11). During infection, the concentrations of Cu^2+^, Fe^2+^, and Zn^2+^ inside phagosomes infected with intracellular pathogens increase between 1 and 24 h postinfection (3, 12), until antibacterial levels of Cu^+^ and Zn^2+^ are reached (8). High concentrations of Cu^+^ generates a ROS response via oxidative stress, protein denaturation, inactivation of enzymes by substitution of other metal cofactors, and membrane destabilization (3, 13, 14). Zn^2+^, unlike Cu^+^, is a redox-inactive nutrient metal. It can bind to nonhomologous proteins at high concentrations, then prevents the protein from obtaining the required metal ion and cause protein dysfunction (15, 16). For example, zinc competitively inhibits manganese binding to an importer in S. pneumoniae and the glycolytic enzymes phosphofructokinase and glyceraldehyde-3-phosphate dehydrogenase in S. pyogenes (17–19). In response, bacteria have evolved elegant strategies, including extensive transporters, transcription factors, and ligands, to counteract the host defense (3, 20–22). Until now, three types of zinc export systems that protect cells from high concentrations of zinc have been identified: RND multidrug efflux transporters, P-type ATPases, and cation diffusion facilitators (CDF) (23, 24). In addition, zinc homeostasis is achieved by export systems and uptake systems which are regulated by zinc-responsive regulator ZntR and zinc uptake repressor Zur, respectively (23, 25). In the presence of zinc, the ion stimulates the DNA-binding activity of these metal regulators, then Zur represses the expression of zinc uptake systems genes and ZntR stimulates the export systems genes expression to lower the intracellular zinc concentration and contributes to the bacterial growth (23).

Mtb and Mycobacterium bovis, two of the well-known intracellular pathogens, have develop mechanisms to adapt to the harsh cytoplasm environment such as zinc toxicity (26, 27). These pathogens contain one putative CDF transporter and 12 P-type ATPase which contain seven P_1B_-type heavy metal-transporting ATPases (CtpA, CtpB, CtpC, CtpD, CtpG, CtpJ, and CtpV) to respond to metal toxicity (3, 28, 29). Among them, the P_1B_-type ATPase CtpC is required for the zinc efflux and optimal intracellular growth of Mtb in human macrophage during infection (30, 31). Several transcription factors, including Zur and the oxidation sensor CmtR, regulate the expression of zinc uptake systems genes such as the *esx*-3 operon to maintain the intracellular zinc homeostasis (32, 33). However, little is known about zinc efflux regulator in mycobacteria. Moreover, CtpC trends to transport Mn^2+^ but not Zn^2+^ under normal experiment condition (34). Therefore, the specific zinc exporter as well as the corresponding molecular mechanisms and signaling pathways of mycobacteria resistance to zinc toxicity are yet to be elucidated.

In this study, we report P_1B_-type ATPase CtpG is a novel zinc exporter in M. bovis BCG and contributes to the mycobacterial resistance to zinc toxicity given that the *ctp*G-deleted strain accumulated more content of free zinc and more hyper-susceptible to physiological concentrations of zinc than that of the wild-type strain. By using the transcriptomic analysis, we identified that zinc induced the expression of *cmt*R operon genes, including *ctp*G and its regulatory gene *cmt*R, and *ctp*G expression induced by zinc depended on transcription factor CmtR. Disruption of CtpG APC domain impaired its ATPase activity, and inhibited zinc efflux and mycobacterial growth under zinc treatment. During infection, CtpG inhibited zinc accumulation of M. bovis BCG, and enhanced the bacterial survival in human macrophages THP-1 and mice with elevated inflammatory responses. Our findings reveal the existence of a novel zinc-triggered signaling circuit, provide new insights into mycobacterial adaptation to the host environment during infection, and might be useful targets for the treatment of tuberculosis.

## RESULTS

### Zn^2+^ broadly affects gene expression of M. bovis BCG.

**FIG 1:**
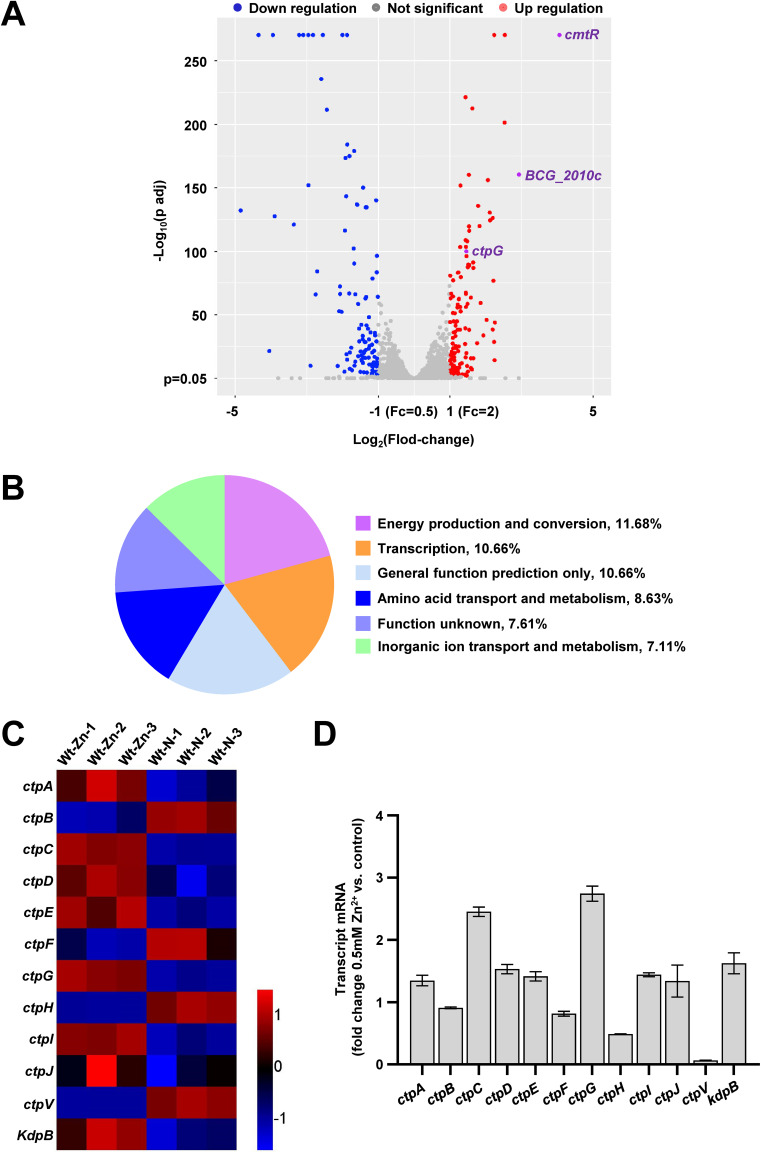
RNA-seq assays for studying the effect of zinc on gene expression in M. bovis BCG. (A) Volcano plot of the difference in gene expression of M. bovis BCG under 0.5 mM zinc treatment determined by RNA-Seq assays. The *x* axis and *y* axis indicate the log_2_(Fold change) values and the log_10_ (adjusted *P* value) values, respectively, of all genes. The Cuffdiff program was executed to perform differential expression tests using the edgeR package. The differential expression of a gene is identified as significant if the fold change is ≥ 2 and the false discovery rate-adjusted *P* value < 0.05. The significantly upregulated and downregulated genes are indicated by red and blue spots, respectively. Genes that did not undergo significant changes in expression are indicated by gray spots. The *cmt*R operon genes included *ctp*G, *BCG_20*10c, and *cmt*R are marked in *pur*ple spots. (B) Function classification of differentially expressed genes (DEGs) mentioned in (A) in the context of Cluster of Orthologous Groups (COG) categories. COGs from each category were normalized to represent the percent abundance of each category to all COGs. 182 of 240 DEGs were assigned to COG classifications and functionally classified into 19 protein families of which the top six groups in terms of abundance ratio are showed in the figure. (C-D) Heat map (C) and transcriptional profile analysis (D) of *ctp* genes expression profile of M. bovis BCG strains determined by the RNA-Seq assays. Red-blue density display showing the expression levels. Wt-Zn-1, Wt-Zn-2, and Wt-Zn-3 represent three biological replicates of differentially expressed genes in the wild-type strain under 0.5 mM zinc treatment, respectively. Wt-N-1, Wt-N-2, and Wt-N-3 represent separately three biological replicates of the genes in the wild-type strain under normal condition.

Zinc is an essential microelement for almost all organisms, but it is toxic at high concentrations, and thus represents an antibacterial mechanism of macrophages. It is really true for intracellular pathogens such as M. tuberculosis (Mtb) and M. bovis. Although these pathogens have developed several mechanisms to adapt to zinc toxicity, the specific zinc exporter and the corresponding signaling pathway for zinc detoxification are yet to be elucidated. For this purpose, we performed RNA-seq and transcriptomic assays to compare the differences in gene expression of M. bovis BCG strains under 0.5 mM zinc treatment. As shown in Fig. 1A, a total of 240 genes were found to be significantly differentially expressed, of which 131 were upregulated and 109 were downregulated. The differentially expressed genes (DEGs) were analyzed using the Cluster of Orthologous Groups (COG) database to classify them and predict their functions. 182 of 240 DEGs were assigned to COG classifications and functionally classified into 19 protein families of which the top six groups in terms of abundance ratio are showed in Fig. 1B. The cluster predicted for energy production and conversion (11.68%) emerged as the largest group, followed by transcription (10.66%), general function prediction only (10.66%), and amino acid transport and metabolism (8.63%). Notablely, 7.11% DEGs are involved in inorganic ion transport and metabolism. Interestingly, inorganic ion transport and metabolism & energy production and conversion are related to metal export, so we focused on the changes in transcript levels of 12 P-type ATPase genes. Indeed, the expression of *ctp*C and *ctp*G was induced under zinc treatment (Fig. 1C and D), which is consistent with previous reported data (30). Therefore, these results indicate that zinc broadly affects genes expression in M. bovis BCG.

### Zn^2+^ remarkably induces the expression of *ctp*G in M. bovis BCG.

**FIG 2:**
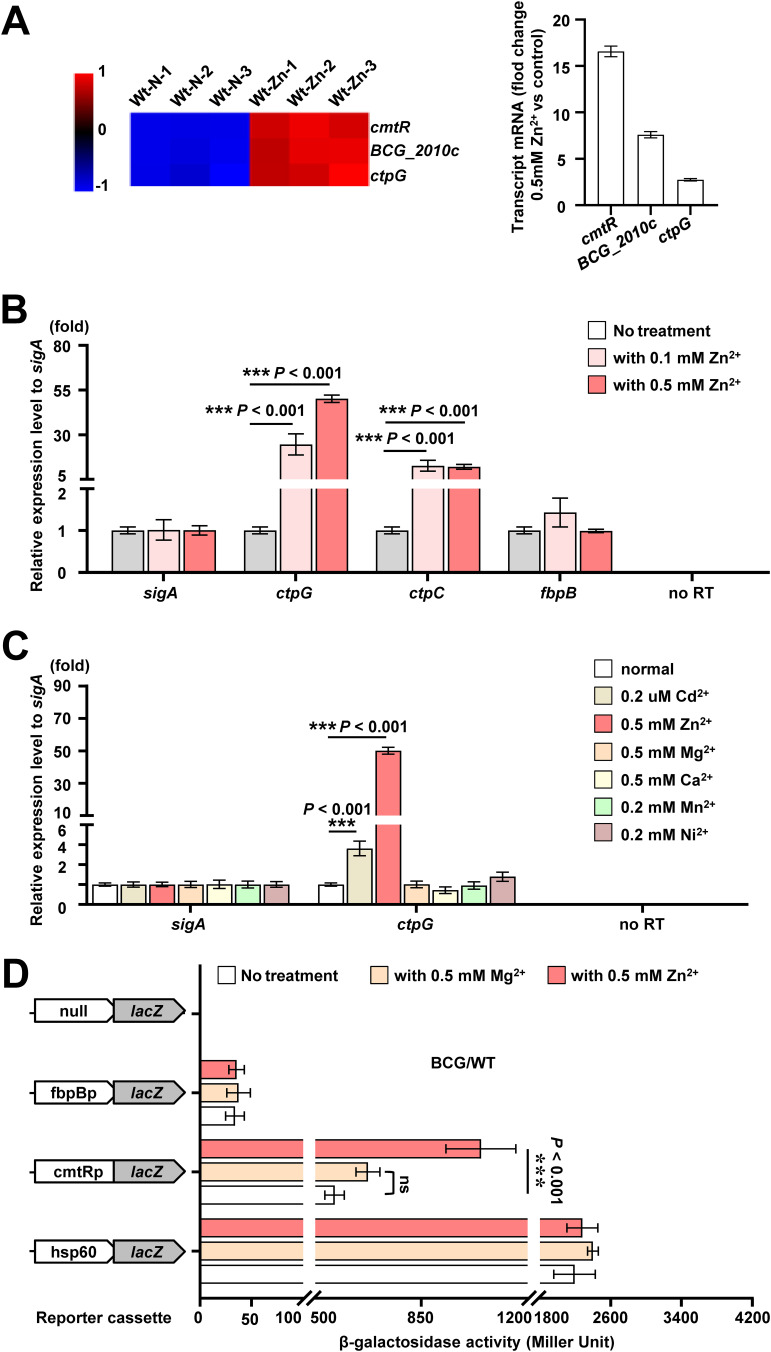
Analysis of the effect of zinc on *ctpG* expression in M. bovis BCG. (A) Heat map and transcriptional profile analysis of *cmt*R operon genes expression profile in the RNA-Seq assays mentioned in Fig. 1. Red-blue density display showing the expression levels. (B-C) RT-qPCR assays for *ctp*G expression of M. bovis BCG under zinc treatment (B) or following the exposure of the mycobacterial strain to various divalent metal cations (C). Bacteria were left untreated or were incubated with 0.5 mM Zn^2+^, 0.2 μM Cd^2+^, 0.5 mM Mg^2+^, 0.5 mM Ca^2+^, 0.2 mM Mn^2+^ or 0.2 mM Ni^2+^ in 7H9 medium for 24 h. (D) Assays for the *cmt*R operon promoter activities under Zn^2+^ or Mg^2+^ treatment. β-gal activity was evaluated in wild-type strain of M. bovis BCG. Left column: schematic representation of recombinant strain generation using reporter plasmids. Null promoter-*lac*Z, hsp60p*-la*cZ, and fbpBp*-la*cZ were used as controls. *Err*or bars represent the S.D. from three biological experiments. The *P* values of the data were calculated by unpaired two-tailed Student's t test using GraphPad Prism7. Asterisks denote significant difference (***, *P* < 0.001, two-tailed Student's t test) between two groups.

Among DEGs mentioned above, we found the upregulation fold of transcription factor *cmt*R was the highest (∼17 times), and the expression of *cmt*R operon genes (Fig. S1A) included *ctp*G was significantly upregulated (Fig. 2A). And CtpG is a P_1B_-type ATPase (Fig. S1B), which indicates that CtpG is implicated in zinc detoxification. To test this hypothesis, we first assayed *ctp*G expression profile of M. bovis BCG and M. smegmatis under 0.1 mM and/or 0.5 mM zinc condition by RT-qPCR, respectively. As shown in Fig. 2B, the induction of *ctp*G expression was enhanced with the increase of zinc concentration in M. bovis BCG, but the expression of *ctp*G_Ms_ was not induced by 0.5 mM zinc in M. smegmatis (Fig. S2A). Next, we compared c*tp* genes expression of M. bovis BCG under 0.5 mM zinc treatment by RT-qPCR assays. As shown in Fig. S2B, *ctp*G expression was strongly induced (up to ∼50 times) upon exposure to 0.5 mM zinc and its induction fold change was much higher than that of the reported Zn^2+^-transporter gene *ctp*C (∼8 times), and the expression of *ctp*H and *ctp*V is inhibited by zinc while other c*tp* genes were not obviously affected under the same experiment conditions, which is consistent with the transcriptome data described above. These results indicate that CtpG may be a main zinc efflux protein in M. bovis BCG. To investigate whether the induction expression of *ctp*G by zinc has specificity, we treated M. bovis BCG strains with different kinds of divalent cations. As shown in Fig. 2C, *ctp*G expression was induced by Zn^2+^ and the control metal Cd^2+^, and the induction effect of Zn^2+^ was much better than that of Cd^2+^. Then, we further confirmed the zinc induction effect by β-gal activity assays. As shown in Fig. 2D, hsp60p significantly promoted the expression of *lac*Z in M. bovis BCG strains relative to the nonpromoter *lac*Z plasmid, which indicates that the reporting system functioned properly. Compared with no treatment, Zn^2+^ instead of Mg^2+^ significantly stimulated the expression of *lac*Z when the *ctp*G promoter, cmtRp, used as a promoter. In contrast, no significant difference was observed in the expression of *lac*Z under different experiment conditions when a negative-control fbpBp was used as a promoter. Collectively, these results suggest that zinc remarkably stimulates *ctp*G expression in M. bovis BCG.

### CtpG enhances the resistance of M. bovis BCG to Zn^2+^ toxicity.

**FIG 3:**
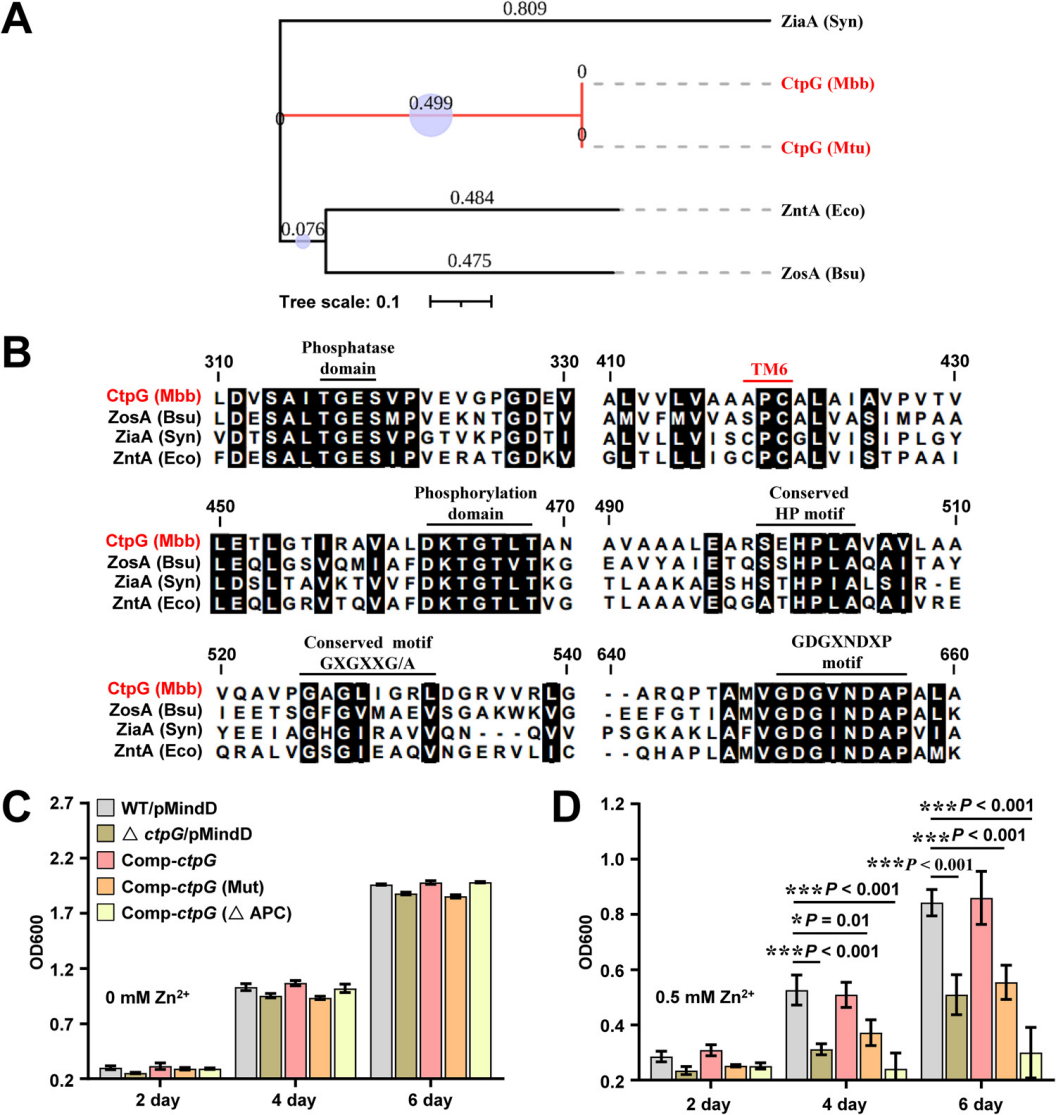
Assays for studying the effect of CtpG on zinc detoxification in M. bovis BCG strains. (A) Phylogenetic relationships of CtpG paralogs. Phylogeny was constructed using the MEGA X software involving the Neighbor-Joining method (bootstrap: 10000 replicates, bootstrap values indicated by circle sizes). CtpG of Mycobacterium bovis BCG and Mycobacterium tuberculosis H37Rv are colored in red. Bacterial sequences shown are from P-type ATPase database (http://traplabs.dk/patbase/): CtpG from Mycobacterium bovis BCG (Mbb) and Mycobacterium tuberculosis H37Rv (Mtu); ZosA from Bacillus subtilis (strain 168) (Bsu); ZiaA from *Synechocystis* sp (Syn); ZntA from Escherichia coli (strain K-12) (Eco). (B) Amino acid sequence alignment (generated using ClustalW) of CtpG and Zn^2+^-ATPases mentioned in (A) for the analysis of conservative amino acid sequences. (C-D) Assays for the effect of *ctp*G deletion on the growth of M. bovis BCG strain in 7H9 medium (C) or medium supplemented with 0.5 mM zinc(D). WT/pMindD represents the BCG/pMindD strain; Δ*ctp*G/pMindD represents the BCG *ctp*G::*hyg*/pMindD strain; comp-*ctp*G represents the BCG *ctp*G::*hyg*/pMindD-*ctp*G strain; comp-*ctp*G (Mut) represents the BCG *ctp*G::*hyg*/pMindD-*ctp*G (Mut) strain, and comp-*ctp*G (ΔAPC) represents the BCG *ctp*G::*hyg*/pMindD-*ctp*G (ΔAPC) strain. *Err*or bars represent the S.D. from three biological experiments. The *P*-values of the data were calculated by unpaired two-tailed Student's t test using GraphPad Prism7. Asterisks denote significant difference (*, *P* < 0.05; ***, *P* < 0.001, two-tailed Student's t test) between two groups.

Next, we performed the phylogeny analysis of CtpG homologs. As shown in Fig. 3A, CtpG are phylogenetically related to Zn^2+^/Cd^2+^ transporters (ZosA and ZntA) (data from P-TYPE ATPase DATABASE). Many conserved sites in these proteins such as phosphatase domain, conserved HP and GXGXXG/A as well as GDGXNDXP motif are highly similar, but the metal-coordinating transmembrane residues in TM6 of CtpG are the APC residues (Ala-Pro-Cys^420^) other than the SPC and CPC residues in the P-type ATPases mentioned above (Fig. 3B). Thus, we speculate that CtpG is a novel protein involved in zinc transport and APC residues play an important role in this process. To confirm this, the cysteine of the APC motif in the sixth TM segment of CtpG was changed to Ala (the APA mutant) and the motif was deleted (ΔAPC) as well. Then, we via an integrative vector constructed wild-type strain (WT/pMindD), *ctp*G-deleted strain (Δ*ctp*G/pMindD), and complementary strains (comp-*ctp*G, comp-*ctp*G [Mut], comp-*ctp*G [ΔAPC]) in M. bovis BCG (Fig. S3 and S4), and assayed their resistance to Zn^2+^/Cd^2+^ toxicity. As shown in Fig. 3D, the growth of Δ*ctp*G/pMindD, comp-*ctp*G (Mut), comp-*ctp*G (ΔAPC) cultured in liquid medium under 0.5 mM zinc were much slower than WT/pMindD for 4 and 6 days. However, no obvious growth difference was observed among these strains in the absence of zinc stress (Fig. 3C). Interestingly, 10 nM Cd^2+^ result in a similar phenotype (Fig. S5A and S5B), which is consistent with the previous results (35). Furthermore, we observed the growth difference among these strains when they were diluted and spotted on 7H10 solid medium containing 0.5 mM zinc. As shown in Fig. S6 (left panel), no growth difference was observed among these strains in medium under normal conditions. In contrast, Δ*ctp*G/pMindD and comp-*ctp*G (Mut) grew worse than that of the WT/pMindD under 0.5 mM zinc treatment, and comp-*ctp*G (ΔAPC) did not grow; no growth difference was observed between comp-*ctp*G and WT/pMindD under the same experiment condition (Fig. S6, right panel). These results indicate that *ctp*G expression enhances M. bovis BCG resistance to zinc toxicity and APC residues play an important role in this process.

### The induction of *ctp*G by zinc depends on CmtR.

**FIG 4:**
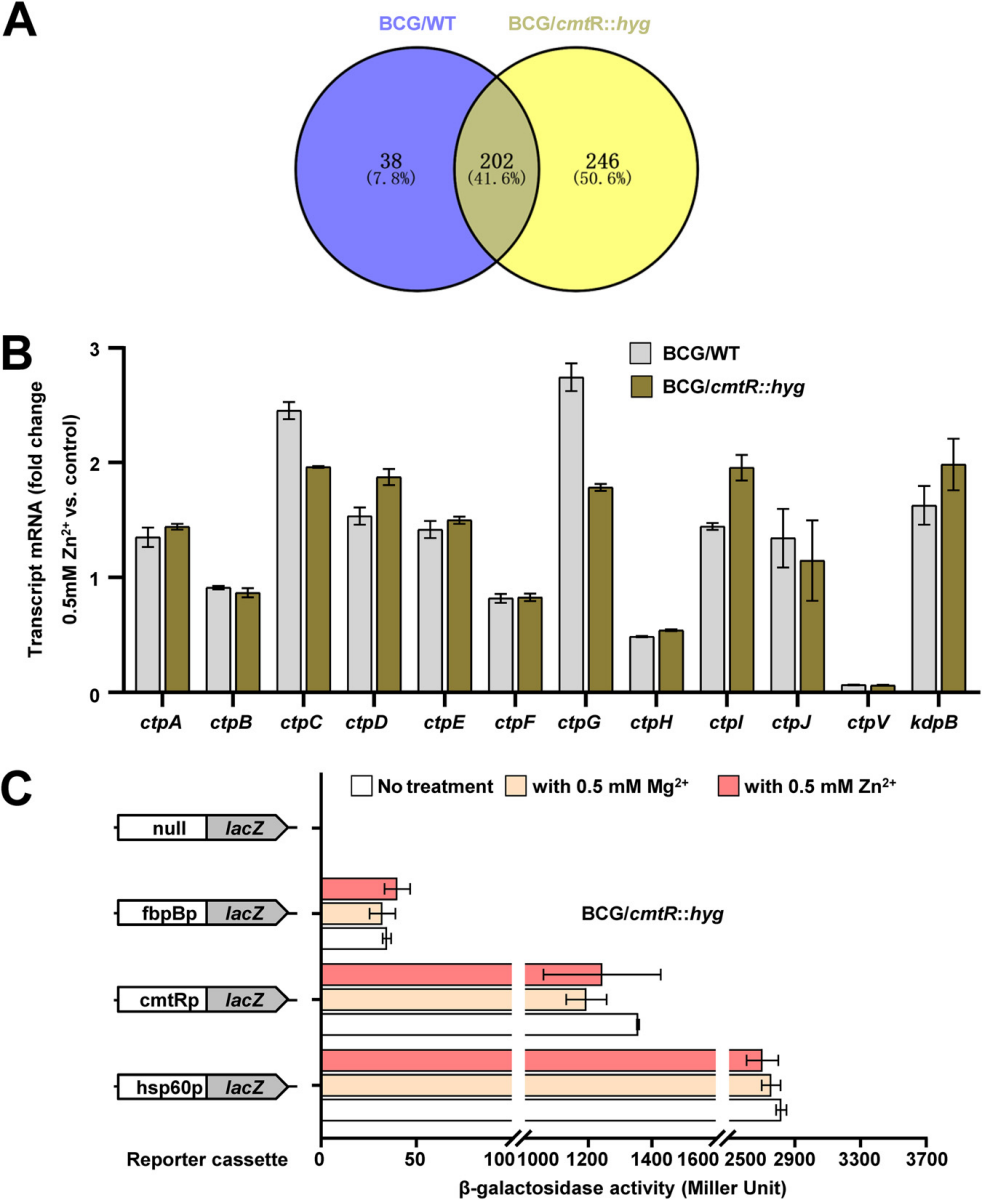
Assays for the regulatory effects of transcription factor CmtR on *ctpG* expression under zinc treatment. (A) Intersectioning the differentially expressed genes in the wild-type and *cmt*R-deleted M. bovis BCG strains under 0.5 mM zinc treatment determined by RNA-Seq assays. (B) Comparative analysis of P-type ATPase genes expression profile of the wild-type and *cmt*R-deleted M. bovis BCG strains using the RNA-seq data. (C) Assays for the promoter activities of cmtRp in the presence or absence of zinc. β-gal activity was evaluated in *cmt*R-deleted strain under 0.5 mM Zn^2+^ or 0.5 mM Mg^2+^ treatment. Left column: schematic representation of recombinant strain generation using reporter plasmids. Null promoter-*lac*Z, hsp60p*-la*cZ, and fbpBp-*lac*Z were used as controls. *Err*or bars represent the S.D. from three biological experiments. The *P*-values of the data were calculated by unpaired two-tailed Student's t test using GraphPad Prism7. Asterisks denote significant difference between two groups using two-tailed Student's t test.

The expression of *cmt*R operon where *ctp*G is located was significantly induced by zinc which can inhibit the DNA-binding ability of CmtR to its promoter (32). Therefore, we assume that zinc-induced *ctp*G expression is dependent on CmtR. To confirm this, we performed RNA-Seq and transcriptomic assays to compare the differential gene expression profile of *cmt*R-deleted strains in M. bovis BCG under 0.5 mM zinc treatment, and then analyzed the correlation of the differentially expressed genes in wild-type strains and *cmt*R-deleted strains. As shown in Fig. 4A (and 4B & Table S1), 38 genes, including *ctp*G, depended on CmtR to respond to zinc. Further, we verified the dependence between *ctp*G expression and CmtR under 0.5 mM zinc by RT-qPCR and β-gal activity assays. As shown in Fig. S7, the expression of *ctp*G and the control gene *f*bpB was remained unaffected in the *cmt*R-deleted strain under 0.5 mM zinc treatment compared with that under no treatment, but *ctp*C expression was significantly induced; no obvious difference was observed in the expression of *lac*Z when cmtRp as well as control promoter fbpBp used as the promoter in *cmt*R-deleted strain under 0.5 mM Zn^2+^ or 0.5 mM Mg^2+^ (Fig. 4C). Taken together, these data indicate that *ctp*G expression induced by zinc is dependent on CmtR.

### Zn^2+^ stimulates the ATPase activity of CtpG.

**FIG 5:**
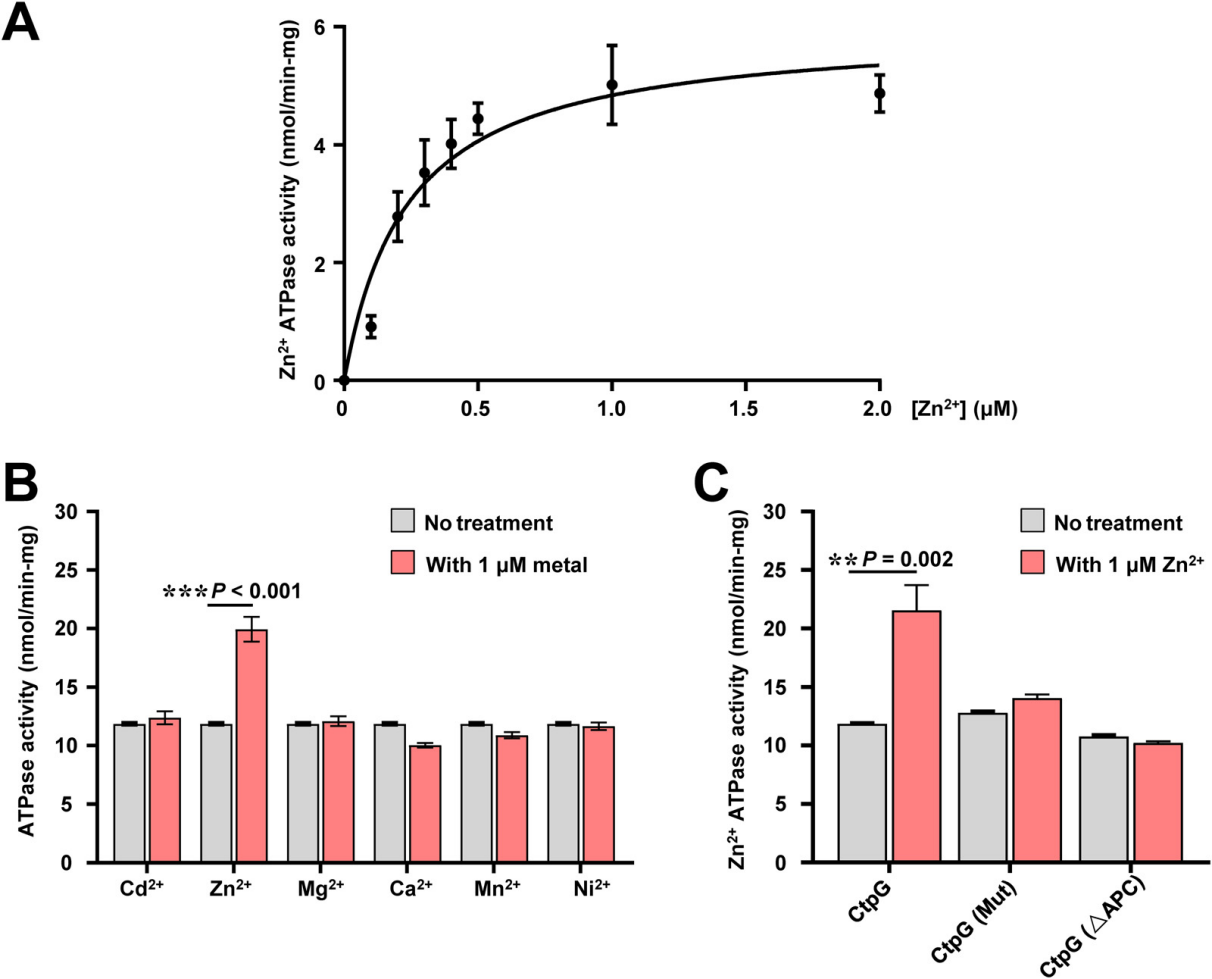
Assays for the effect of zinc on the ATPase activity of CtpG. (A) Kinetic parameters of CtpG. The Zn^2+^-ATPase activity of CtpG follows a Michaelis-Menten kinetics. Zinc was added at different concentrations (0.1 to 2.0 μM) and the released phosphate (Pi) from the hydrolysis of ATP was quantified in the enzymatic reactions. The kinetic parameters for CtpG (*K*_1/2_ 0.24 ± 0.05 nM and *V_ma_*_x_ 6 ± 0.4 nmol/mg min) were calculated using GraphPad Prism 7. Error bars represent the S.D. from three biological experiments. (B) The ATPase activity of CtpG in the presence of 1 μM metals. The enzymatic ATPase activity corresponds to nmol of Pi released/mg of protein. min. (C) Assays for the effect of zinc on the ATPase activity of CtpG (Mut) and CtpG (△APC). The purified proteins CtpG, CtpG (Mut) and CtpG (△APC) were incubated with or without 1.0 μM zinc and measured the ATPase activity. Error bars represent the S.D. from three biological experiments. The *P*-values of the data were calculated by unpaired two-tailed Student's t test using GraphPad Prism7. Asterisks denote significant difference (**, *P* < 0.01; ***, *P* < 0.001, two-tailed Student's t test) between two groups.

CtpG belongs to the family of P_1B_-ATPase which follows the classical E1/E2 Albers-Post transport of metals across the membrane. The central characteristic of the transport mechanism of all P-ATPases is coupling of transmembrane substrate transport to ATP hydrolysis (28). Therefore, we cloned and expressed CtpG, CtpG (Mut) and CtpG (ΔAPC) in E. coli (Fig. S8A), then assayed their ATPase activity. As shown in Fig. S8B, the ATPase activity increased parallel to the amount of these proteins that were added to the enzymatic reaction, and no significant activity difference among these proteins was observed. Then, we verified the effect of zinc on the ATPase activity of CtpG. As shown in Fig. 5A, the ATPase activity of CtpG was stimulated by addition zinc into the assay media, and the dependence of the ATPase activity on the zinc concentration showed an apparent *K_1/_*_2_ of 0.24 ± 0.05 μM, a *V*_max_ of 6 ± 0.4 U/mg. Furthermore, we evaluated metal ion specificity for the ATPase activity of CtpG. As shown in Fig. 5B, CtpG exhibited the maximum ATPase activity in the presence of zinc compared to other divalent metal cations when 1 μM metal ion was used, which indicates that zinc specifically stimulated the ATPase activity of CtpG. Based on this result, we compared the ATPase activity difference of CtpG, CtpG (Mut), and CtpG (ΔAPC). As shown in Fig. 5C, the ATPase activity of CtpG was significantly increased in the presence of 1 μM zinc, but the ATPase activity of CtpG (Mut) and CtpG (ΔAPC) were not affected. Collectively, these results indicate that zinc effectively promotes the ATPase activity of CtpG in M. bovis BCG and the APC residues play an important role in the process.

### CtpG promotes Zn^2+^ efflux of M. bovis BCG.

**FIG 6:**
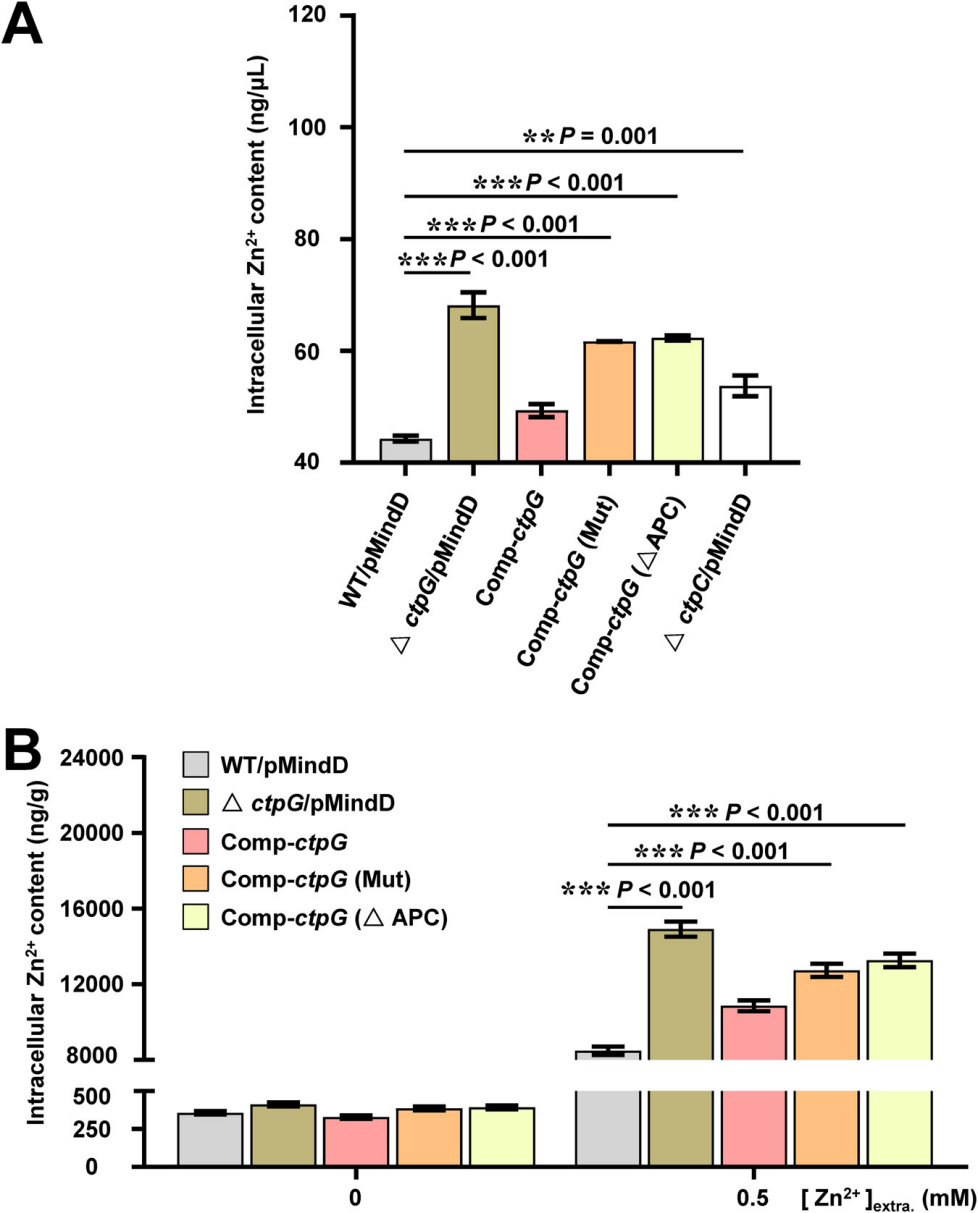
Assays for the effect of CtpG on zinc efflux in M. bovis BCG strains. (A) Zinc assay kit analysis of the metal accumulation in bacterial cells under 0.5 mM zinc treatment. Δ*ctp*C/pMindD represents the BCG *ctp*C::hyg/pMindD strain. (B) ICP-OES assays for the effect of CtpG on zinc accumulation of M. bovis BCG strains under 0.5 mM zinc treatment. Recombinant strains (mentioned in Fig. 3) was left untreated or incubated with 0.5 mM zinc for 24 h at 37°C, washed twice in PBS, and the bacterial pellets processed for ICP-OES analysis. Intrabacterial zinc content expressed as nanograms zinc per grams of bacterial extract. Error bars represent the S.D. from three biological experiments. The *P*-values of the data were calculated by unpaired two-tailed Student's t test using GraphPad Prism7. Asterisks denote significant difference (**, *P* < 0.01; ***, *P* < 0.001, two-tailed Student's t test) between two groups.

When P_1B_-ATPase binds the transported metal, the enzyme undergoes phosphorylation, followed by a conformational change, which expels the metal from the cytoplasm (28). Here, we observed zinc promotes the ATPase activity of CtpG which contributes to mycobacterial resistance to zinc toxicity, implying that CtpG plays an important role in zinc export. To confirm this, we constructed a deletion strain of the reported zinc transporter CtpC in M. bovis BCG (Fig. S4), and treated wild-type strain, *ctp*C-deleted strain, *ctp*G-deleted strain, and complementary strains mentioned above with 0.5 mM zinc for 24 h, then assayed their intracellular zinc accumulation. As shown in Fig. 6A, zinc assay kit analysis revealed that the intracellular zinc content in Δ*ctp*G/pMindD, comp-*ctp*G (Mut), comp-*ctp*G (ΔAPC), and Δ*ctp*C/pMindD was much higher than that in WT/pMindD under 0.5 mM zinc treatment; interestingly, the intracellular zinc content in Δ*ctp*G/pMindD is also obviously higher than that in Δ*ctp*C/pMindD. These results indicate that CtpG may be the main zinc exporter in M. bovis BCG and its APC residues are essential for zinc efflux. To further confirm this, we assayed the intracellular zinc content in these strains under the same experiment conditions by ICP-OES. As shown in Fig. 6B, the intracellular zinc content in Δ*ctp*G/pMindD, comp-*ctp*G (Mut), and comp-*ctp*G (ΔAPC) were significantly higher than that in WT/pMindD under 0.5 mM zinc treatment. In contrast, no obvious difference was observed among these strains under normal conditions. Taken together, these results indicate that CtpG facilitates excess zinc export and the APC residues play a pivotal role in the process.

### CtpG enhances M. bovis BCG resistance to Zn^2+^ toxicity and enhances mycobacterial survival in THP-1 cells.

**FIG 7:**
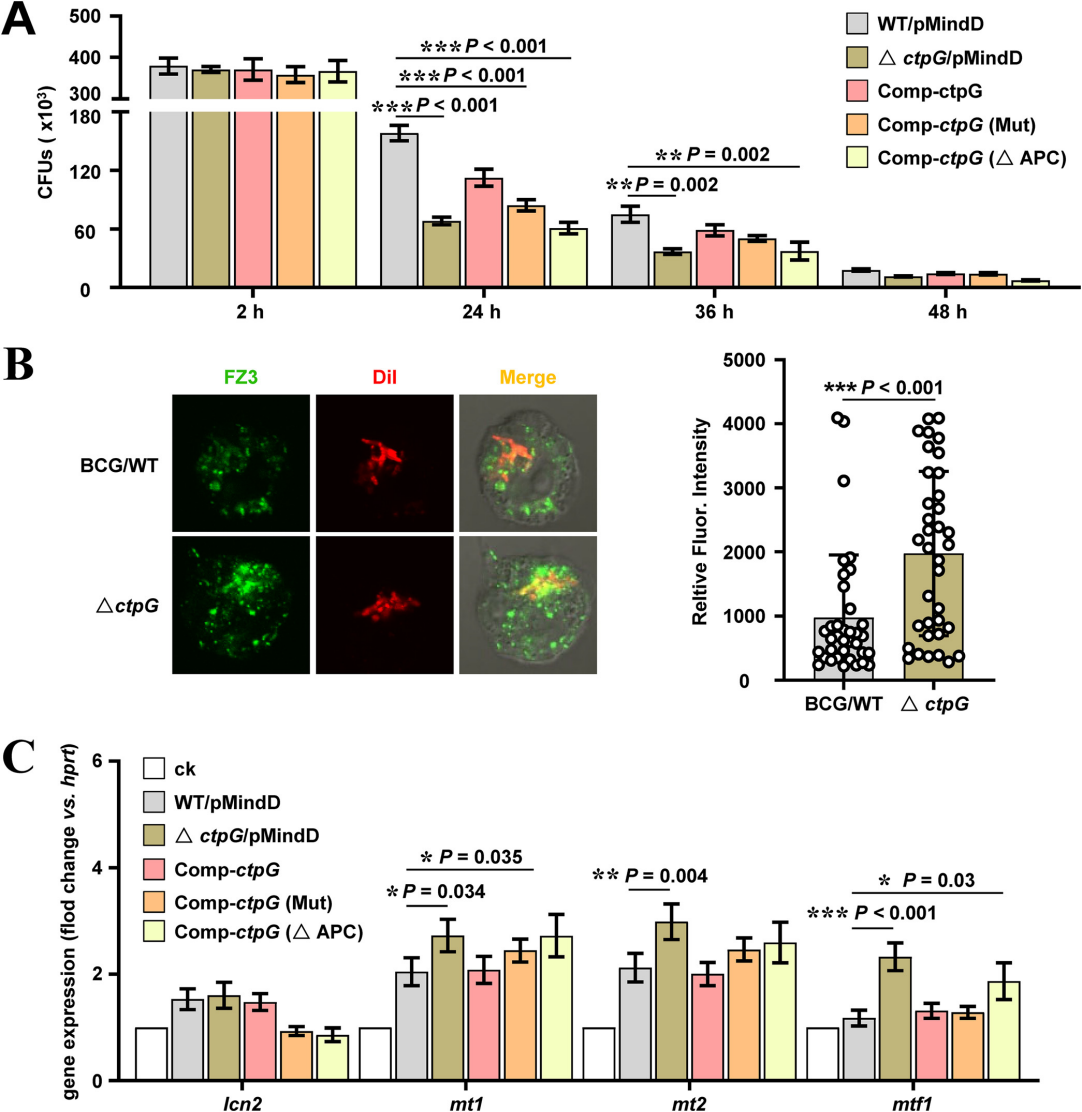
Assays for the effects of CtpG on mycobacterial survival and zinc accumulation in THP-1 macrophages. (A) Intracellular survival of M. bovis BCG strains in macrophages. Recombinant strains (mentioned in Fig. 3) were used to infect THP-1 macrophages at a multiplicity of infection of 10 mycobacteria per cell. After 4 h, cells were washed and incubated in fresh medium, then and lysed at the indicated postinfection time points. Serial dilutions of the supernatant were plated on 7H10 agar supplemented with 10% oleic acid-albumin-dextrose-catalase, and the number of CFU was counted 15–21 days later. (B) Composite images and quantification of DiI-labeled M. bovis BCG strains (in red), FluoZin-3 (FZ3)-labeled free zinc (in green) in the THP-1 macrophages after 20 h infection. Data points are the mean relative fluorescence of bacterial intracellular zinc from at least 35 FZ3-positive phagosomes in the graph left panel. (C) RT-qPCR analysis of the expression of the genes (*lcn*2, mt1, *mt2*, and *mtf*1) at 20 h after M. bovis BCG strains infection in THP-1 macrophages. Error bars represent the S.D. from three biological experiments. The *P*-values of the data were calculated by unpaired two-tailed Student's t test using GraphPad Prism7. Asterisks denote significant difference (*, *P* < 0.05; **, *P* < 0.01; ***, *P* < 0.001, two-tailed Student's t test) between two groups.

Excess zinc represents an antimicrobial strategy for macrophages, and CtpG enhances M. bovis BCG resistance to the stress, which implies that CtpG contributes to bacterial survival in the host cells. To confirm this, we utilized THP-1 cell model to evaluate the effect of CtpG on the intracellular survival of M. bovis BCG. The results indicated that these strains invaded macrophages comparably at 2 h postinfection (hpi). However, the intracellular survival efficiency of Δ*ctp*G/pMindD, comp-*ctp*G (Mut), and comp-*ctp*G (ΔAPC) in THP-1 macrophages was significantly lower than that of WT/pMindD at 24 and 36 hpi (Fig. 7A). Similarly, the survival efficiency of Δ*ctp*G/pMV261 was significantly lower than that of WT/pMV261 in THP-1 macrophages at 20 hpi, whereas the survival efficiency of OE-*ctp*G was observably higher than that of WT/pMV261 (Fig. S9). Collectively, these results indicate that CtpG enhances mycobacterial survival in THP-1 macrophages.

Considering that some pathogens such as Mtb and M. bovis are usually exposed to zinc toxicity when infecting macrophages, and CtpG contributes to mycobacterial survival under zinc toxicity and in THP-1 macrophages, we speculate that CtpG enhances mycobacterial resistance to zinc toxicity in the cells. To test this hypothesis, we set up to visualize the intracellular free zinc in THP-1 macrophages with FluoZin-3 (FZ3), a fluorescent probe specific for free zinc, by confocal assays during M. bovis BCG strains infection (36, 37). As shown in Fig. 7B, significantly increased colocalization between *ctp*G-deleted strain and FZ3 in the infected THP-1 macrophages was observed compared with that of the wild-type strain, and the *ctp*G-deleted strain is more likely to induce the production of zinc in macrophages (Fig. S10A). Further, we assayed the expression of several metallothionein (MT)-encoding genes (*mt*1, *mt*2, and *mt*f1) which were induced when the THP-1 cell is exposed to potentially toxic concentrations of free zinc (38). As shown in Fig. S10B, the induction fold of these genes was the most obvious at 20 hpi when THP-1 macrophages were infected with M. bovis BCG. Subsequently, we compared the expression difference of these genes at 20 hpi when recombinant strains infected. As shown in Fig. 7C, Δ*ctp*G/pMindD, comp-*ctp*G (Mut), and comp-*ctp*G (ΔAPC) were significantly induced higher expression of *mt1*, *mt2,* and *mtf*1 than that of WT/pMindD. Collectively, our data suggest that CtpG plays a crucial role in the ability of M. bovis BCG to resist zinc poisoning and enhance mycobacterial survival at least in the context of human macrophages.

### CtpG enhances M. bovis BCG survival in mice.

**FIG 8:**
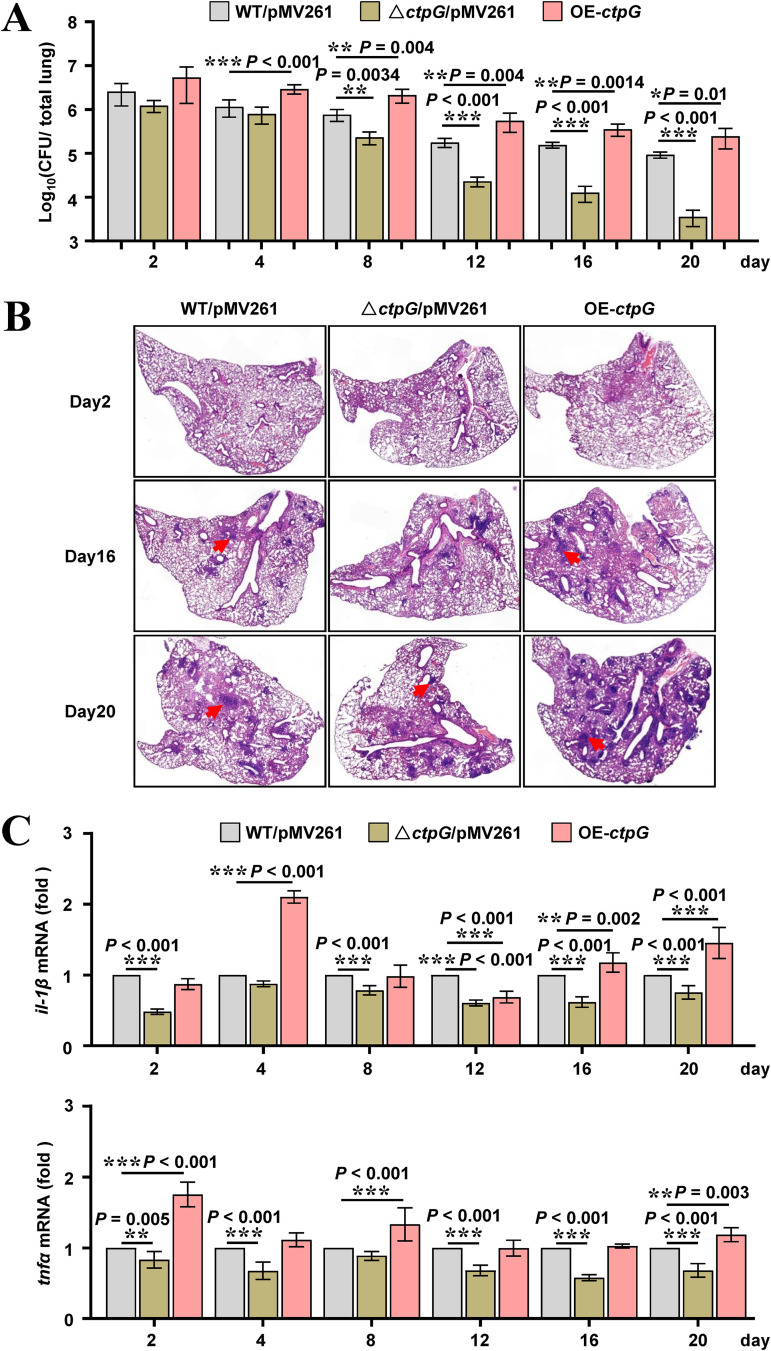
Assays for the effects of CtpG on the host innate immunity responses. (A) Bacterial titers in homogenates of lung from SPF C57BL/6 mice (*n* = 6 mice per group) infected intratracheally with 1.0 × 10^6^ of M. bovis BCG strains for 0–20 days. WT/pMV261 represents the BCG/pMV261 strain; Δ*ctp*G/pMV261 represents the BCG *ctp*G::*hyg*/pMV261 strain; OE-*ctp*G represents the BCG/pMV261-*ctp*G strain. (B) Histopathology of lungs of mice infected as in (A), assessed in sections stained with hematoxylin and eosin. (C) Quantitative PCR analysis of *tnf* mRNA and *il1*b mRNA in splenic cells from mice infected as in (A). Data are representative of one experiment with two independent biological replicates (mean and S.D. of *n* = 6 mice per group). The *P*-values were calculated by unpaired two-tailed Student's t test using GraphPad Prism7. Asterisks denote significant difference (**, *P* < 0.01; ***, *P* < 0.001, two-tailed Student's t test) between two groups.

To investigate whether CtpG affects M. bovis BCG survival in mice, we determined the bacterial loads in the lungs of C57BL/6 mice infected with the wild-type, *ctp*G-deleted, and *ctp*G-overexpressing strains. As shown in Fig. 8A, the bacterial loads in the lung tissues of mice infected with all three strains were similar at 2 days postinfection (dpi); however, the bacterial load in the lungs of mice infected with the *ctp*G-overexpressing strain increased significantly compared with that of mice infected with the wild-type strain from 4 to 20 dpi. In contrast, compared with the mice infected with the wild-type strain, those infected with the *ctp*G-deleted strain exhibited an obvious decrease in bacterial load since 4 dpi, which indicates that CtpG enhances M. bovis BCG survival in the lungs of mice. Furthermore, histopathological assay of lungs showed that mice infected with the *ctp*G-deleted strain had less-severe lung pathology, marked by reduced total cellular and neutrophilic infiltration than those infected with the wild-type strain, probably because the high load of bacteria triggered regulatory immune responses in the host (Fig. 8B and Fig. S11). Consistently, levels of *tnf* mRNA and *il1b* mRNA in mice infected with the *ctp*G-deleted strain were much lower (Fig. 8C). Taken together, these data suggest that CtpG enhances M. bovis BCG survival in mice.

## DISCUSSION

Although zinc is an essential micronutrient for the survival and proliferation of bacteria, it is toxic at high concentrations and presents an antibacterial mechanism for macrophages. Mtb is considered one of the most persistent intracellular pathogens and has developed unique mechanisms to adapt to host environments, including zinc deficiency and excess, during infection (10, 39). However, the molecular mechanism by which mycobacteria promote detoxification of zinc and the associated signaling pathways remains largely unclear. In this study, using M. bovis BCG as a model, we first characterized the P_1B_-type ATPase CtpG acts as a novel zinc efflux protein in which the APC domain plays an important role in zinc transport, and enhances mycobacterial resistance to zinc toxicity. We further observed that CtpG responding to zinc depends on transcription factor CmtR. Lastly, we provided evidence to demonstrate that CtpG affects mycobacterial interaction with host cells and contributes to bacterial survival during infection. Our findings revealed the existence of a novel regulatory pathway on mycobacteria responding to and adapting to host-mediated zinc toxicity.

An interesting finding from the present study is that CtpG is a novel and main zinc efflux protein in M. bovis BCG. It has a highly developmental homology to the zinc transport proteins that have been reported in other species and belongs to a metal transport P_1B_-ATPase with characteristic phosphorylation domains and membrane topology (28, 40). It is now well established that metal specificity is conferred by conserved residues in the TMs flanking the large cytoplasmic ATP binding and hydrolysis domain (34, 41, 42). The analysis of the eight transmembrane sequences of homologous CtpG proteins showed that CtpG has a novel residue APC signature in TM6, and has a unique HEFTE in TM8 (Fig. 3B and Fig. S1B). Furthermore, many experimental evidences indicate that conserved Cys in TM6 participate in metal binding and transport. It has been reported that various sequences in TM6 are related to ion transport and essential for enzyme function, such as CPC, CPH, SPC, TPC or CPS. In addition, the requirement of conserved transmembrane His(H), Glu(E), Asp(D), Ser(S) and Met(M) residues for the metal translocation by various transporters has been established (40). The presence of these unique residues is not trivial because their replacement leads to inactive proteins. Interestingly, these transformations make CtpG highly specific for zinc. Direct biochemical analysis of CtpG activity showed that maximum activation of the transporter occurs in the presence of zinc. What’s more, we found that mutating APC residues to APA residues or delete it can seriously affect the enzymatic activity and zinc efflux of CtpG as well as mycobacterial resistance to zinc toxicity. In addition, we found zinc significantly induces *ctp*G expression, and the induction fold change is higher than that of the previously characterized export transporter CtpC. And the zinc efflux effects of CtpG is significantly better than that of CtpC. Therefore, CtpG may be a main efflux protein for M. bovis BCG adaptation to the zinc poisoning environment.

Zinc homeostasis is crucial for bacterial cells, and too much or too little can result in growth defect. Therefore, intracellular zinc concentration is tightly regulated in the cells. Usually, the process involves pairs of zinc-responsive transcription factors, similar to Zur and ZntR, which separately regulate the zinc uptake and efflux (25, 43). For example, in B. abortus, the expression of the genes encoding the zinc uptake system ZnuABC is negatively regulated by Zur (44). And ZntR controls the expression of the gene encoding the zinc exporter ZntA by binding directly to its promoter to adaptation to zinc toxicity environment (45–47). However, only the zinc uptake repressor Zur have been characterized in mycobacterial species until now. And little is known about the mechanisms and regulatory pathway on zinc efflux in these bacteria. In the present study, we successfully characterized CtpG acts as a novel zinc export transporter in M. bovis BCG and significantly enhances mycobacterial resistance to zinc toxicity. Zinc induces the expression of *ctp*G and promotes the ATPase activity of CtpG. Furthermore, we found the expression of *ctp*G induced by zinc depends on its upstream repressor CmtR which has been reported to regulate zinc homeostasis by interaction with Zur in M. bovis BCG. Therefore, our findings revealed a novel regulatory model for mycobacterial resistance to zinc toxicity.

P-type ATPases make up a ubiquitous family of proteins which couple the hydrolysis of ATP to transport cations across plasma membranes and is pivotal for cell viability. For example, Mtb P_1B_-type ATPase, CtpA, is associated with Cu^+^ transport, and plays an important role in Cu^+^ detoxification (48). Similarly, a putative copper exporter CtpV contributes to maintain mycobacterial resistance to Cu^+^ toxicity and enhance intracellular survive during infection (49). In addition, CtpC has been reported to be a zinc efflux transporter and required for optimal growth of Mtb under excess zinc stress and in human macrophages. However, CtpC does not transport zinc under normal experiment condition and has no significant effect for bacterial survive in mouse. CtpG, like CtpC, belongs to be a P_1B_-type ATPase and transports Cd^2+^ to protect M. smegmatis against metal toxicity (35). In the present study, we found CtpG promotes zinc efflux and enhances mycobacterial resistance to zinc toxicity in M. bovis BCG. Zinc specifically induces *ctp*G expression and stimulates its ATPase activity. Moreover, during infection, CtpG lowers the intracellular zinc accumulation and contributes to mycobacterial survival in human macrophages and in mouse. Therefore, CtpG may represent a novel defense weapon for mycobacteria to survive in host.

In summary, we characterized CtpG acts as a novel zinc efflux transporter and significantly enhances M. bovis BCG survival under excess zinc stress and during infection. Our data combined with that of previous studies suggest that CmtR is able to sense zinc and derepresses *ctp*G expression, following which CtpG binds zinc and hydrolyzes ATP to release energy for intracytoplasmic zinc efflux. Our findings first identified a regulation signaling pathway by transcription regulator CmtR on CtpG-mediated zinc detoxification in mycobacteria and provide insights into stress-induced mycobacterial adaptation to the host environment.

## MATERIALS AND METHODS

### Plasmids, enzymes, and reagents.

The pET28a plasmid obtained from Novagen was used for overexpressing the mycobacterial protein in Escherichia coli BL21(DE3) pLysS strain (Novagen, Darmstadt, Germany). The primers (listed in Table S2) for PCR were synthesized by Tsingke (China); DNA polymerase, restriction enzymes, T4 ligase, dNTPs, and antibiotics were obtained from TaKaRa Biotech (Shiga, Japan). The 7H9/7H10 medium and oleic acid-albumin-dextrose-catalase enrichment used for mycobacterial growth were purchased from BD Biosciences. Wild-type female SPF C57BL/6 mice were purchased from Chang-sheng Bio (Liaoning, China).

### Expression and purification of recombinant proteins.

Genes were amplified using PCR with specific primer pairs (Table S2). Mutant genes were obtained through site-directed mutagenesis by overlapping extension PCR. The amplified DNA fragments were digested using the corresponding restriction endonucleases and cloned into the pET-28a expression vector to produce recombinant plasmids (Table S3). The expression strains of E. coli BL21(DE3) pLysS containing the recombinant plasmids were cultured, and the recombinant proteins were purified, as described in a previous study (50). All purification steps were carried out at 0–4°C. Cells were suspended in buffer A (25 mM Tris, pH 7.0, 100 mM sucrose, 1 mM phenylmethylsulfonyl fluoride [PMSF; Sigma]) and disrupted with a French press at 20,000 lb/in^2^ Lysed cells were centrifuged at 8,000 × *g* for 30 min. The supernatant was then centrifuged at 229,000 × *g* for 1h, and the pelleted membranes were resuspended in buffer A (10–15 mg/mL). For protein solubilization and purification, membranes were diluted to a final concentration of 3 mg/mL in buffer B (25 mM Tris, pH 8.0, 100 mM sucrose, 500 mM NaCl, 1 mM PMSF) and solubilized with 0.75% dodecyl-β-d-maltoside (DDM; Calbiochem). The preparation was incubated for 1 h at 4°C with mild agitation and centrifuged at 229,000 × *g* for 1h. The supernatant was incubated overnight at 4°C with Ni^2+^-nitrilotriacetic acid resin (Qiagen) pre-equilibrated with buffer B, 0.05% DDM, and 5 mM imidazole. The resin was washed with buffer B, 0.05% DDM containing 10 mM and 20 mM imidazole, and the protein was eluted with buffer B, 0.05% DDM, 250 mM imidazole. Fractions were pooled and concentrated, and buffer was replaced by buffer C (25 mM Tris, pH 8.0, 100 mM sucrose, 50 mM NaCl, and 0.01% DDM) using 50 kDa cutoff centricons (Millipore). The proteins were aliquoted and stored in 20% (vol/vol) glycerol at −80°C until use. All protein determinations were performed in accordance with the Bradford method (51). Purified CtpG protein was analyzed by 10% SDS-PAGE followed by Coomassie brilliant blue (CBB) staining.

### Construction of recombinant mycobacterial strains.

*ctp*G, *ctp*G (Mut), *ctp*G (ΔAPC) and *ctp*C genes were amplified by PCR using specific primer pairs (Table S2), and the amplicons were digested by the corresponding restriction endonucleases. The digested *ctp*G, *ctp*G (Mut), *ctp*G (ΔAPC) and *ctp*C genes fragments were separately cloned into pMV261 overexpression vectors (52) or a pMindD vector (53) and transformed into the wild-type and knockout strains of M. bovis BCG to generate overexpression and complementary strains, respectively, in which the expression of the target genes was regulated using anhydro-tetracycline hydrochloride. *ctp*G or *ctp*C knockout was performed in M. bovis BCG strains, as described previously (54).

### Transcriptomic analysis.

*M. bovis* BCG strains (BCG/WT and BCG/*cmt*R::*hyg*) were cultured in 7H9 medium with shaking at 160 rpm at 37°C until the midlogarithmic phase. Then, the cells were treated with 0.5 mM zinc for 24 h and harvested from each sample (each strain in three biological replicates). Subsequent transcriptomic analysis was performed, as described previously (54). In brief, total RNA was isolated using RNAprep Pure Cell/Bacteria kit (Tiangen, China). Strand-specific libraries were prepared using the NEBNext Ultra RNA Library Prep kit for Illumina (Illumina, USA) according to the manufacturer’s instructions. Library construction and sequencing were performed at Novogene Corporation.

The volcano plot diagrams were constructed using the log_10_(*P* value) and log_2_(fold change) values of the genes of M. bovis BCG strains under zinc treatment conditions using the ggplot2 package. Briefly, the cuffdiff program (55) was performed to conduct differential expression tests for the wild-type or *cmt*R-deleted samples under zinc treatment using the edgeR package (56). A transcript will be reported as differential expression significant if the test gives that the false discovery rate-adjusted *P*-value after Benjamini-Hochberg correction for multiple-testing represents statistical significance (*P* value < 0.05) (57) and the fold change is ≥ 2. The changes in gene expression are indicated on the *x* axis, and the *P* values are indicated on the *y* axis. The red and blue spots represent the upregulated and downregulated genes, respectively, whereas the *g*ray spots represent genes with insignificant changes in expression.

The heat map was constructed using the HemI (Heatmap Illustrator, version 1.0) software. Briefly, the fragments per kilobase of exon per million fragments mapped values of target genes in each sample were normalized and imported to the HemI software to construct the heat map diagram. Red represents high expression, whereas blue represents low expression of the target genes in different samples. The color scale beside the heat map indicates the color threshold.

### Assay for β-gal activity.

Assays for measuring β-gal activity was separately performed using the wild-type and *cmt*R-deleted M. bovis BCG strains by constructing operon-*lac*Z fusions based on the expression vector pMV261. The target and control promoters were amplified by PCR using the respective primers, which are listed in (Table S2), after which the amplicons were digested using the corresponding restriction endonucleases and cloned into the pMV261 backbone. The reporter gene *lac*Z was cloned downstream of the promoters. The plasmids were separately transformed into the *cmt*R-deleted and wild-type M. bovis BCG strains to obtain the corresponding recombinant reporter strains. The recombinant strains were cultured until the midlogarithmic phase in 7H9 medium, then treated with 0.5 mM Zn^2+^ and 0.5 mM Mg^2+^ for 24 h, respectively. The bacterial cells were harvested and washed using phosphate-buffered saline (PBS). The levels of galactosidase were measured as described previously (32).

### Evaluation of mycobacterial growth.

The growth patterns of M. bovis BCG strains were evaluated using modified versions of procedures described earlier (32). The recombinant strains were cultured in 7H9 medium supplemented with 30 μg/mL Kan and 50 ng/mL anhydrotetracycline hydrochloride, and the cultures were incubated under shaking conditions at 160 rpm at 37°C. When the culture reached the midlogarithmic phase, each culture was diluted (4:100) in 100 mL of fresh 7H9 broth supplemented with 0.5 mM zinc, and the sensitivity of the recombinant strains was determined every 2 days.

### Determination of metal ion content under zinc treatment.

Total zinc contents in the dried bacterial pellets was determined by ICP-OES (Varian, USA) according to a previously published procedure with several modifications (34). Briefly, the *ctp*G recombinant strains were cultured in 300 mL of 7H9 medium under shaking conditions at 160 rpm at 37°C until the midlogarithmic phase, after which the strains were treated with 0.5 mM zinc for 24 h. The harvested samples were washed twice in PBS containing 1 mM EDTA and 0.05% Tween 80, and were then washed only with PBS. The pellets were stored overnight at −80°C and dried using the Freeze Dryer machine (Thermo Fisher). The dried samples were weighed and acid digested with HNO_3_ (trace metal grade) for 4 h at 80°C and overnight at 65°C. The supernatants were filtered by passing through 0.22-μm filters (Corning Inc.). The metal content in the digested samples was measured by ICP-OES. The concentration of zinc in the *ctp*G recombinant strains were further determined using a Zinc assay kit (MAK032; Sigma-Aldrich). Briefly, M. bovis BCG strains were grown to midlogarithmic phase, were pelleted, washed, and suspended in PBS, and were ultrasonic crushing treatment 10 min (200 W). The extract was deproteinized by adding an equal volume of trichloroacetic acid (7%). The mixture was then centrifuged at 12000 rpm for 5 min to obtain the cell extract as the supernatant. Then, 50 μL of the cell extract was mixed with 200 μL of Zn reagent mix and the reaction mixture was incubated at room temperature for 10 min. Absorbance of the reaction mixture was read at 560 nm.

### ATPase assays.

The enzymatic reactions (50 μL) were performed in the reaction buffer (40 mM MOPS-TRIS, 3 mM MgCl_2_, 5 mM NaN_3_, 0.25 mM Na_2_MoO_4_, and 0.5 mM Cys, pH 7.0) using 4 μg of CtpG protein, and supplemented with 1 μM (final concentration) of the tested heavy metal cation (Cd^2+^, Zn^2+^, Mg^2+^, Ca^2+^, Mn^2+^ and Ni^2+^). The enzymatic reactions were initiated by the addition of 3 mM Na_2_ATP, followed by incubation at 37°C for 30 min. The ATPase activity was stopped by adding 100 μL of stop solution (3% ascorbic acid, 0.5% ammonium molybdate, and 3% SDS in 1.0 M HCl) and 150 μL of 3.5% bismuth citrate and 3.5% sodium citrate in 2.0 M HCl. Subsequently, the released inorganic phosphate (Pi) was quantified (58). The OD_690_ of the samples was measured using an iMark. Microplate Absorbance Reader (Bio-Rad, Philadelphia, PA, United States) and interpolated in a calibration curve from 0 to 100 nM NaH_2_PO_4_. The total enzymatic activity was reported as nmol of Pi released/mg of protein. min; the reactions were assessed in triplicate from three independent experiments.

### Kinetic parameters of CtpG.

The Zn^2+^-ATPase activity of CtpG was assayed as described above (50). To estimate the dependence of CtpG on zinc concentration, enzymatic kinetics were performed under the optimal reaction conditions and varying the zinc concentration from 0.1 to 2 μM and the enzymatic reactions were performed for 30 min. The enzymatic activity was reported as nM of Pi released/mg of protein. min and assessed in triplicate for three independent experiments. The values of *K_1/_*_2_ and *V_ma_*_x_ were calculated using the nonlinear least-squares regression for Michaelis-Menten enzyme kinetics using Prism 7 version, GraphPad Software, La Jolla California USA.

### Intracellular survival assays.

Monocytic THP-1 cells were seeded in 24-well plates (5 × 10^5^ cells per well in RPMI 1640 supplemented with 10% heat-inactivated fetal bovine serum (FBS) (Gibco) in a volume of 1 mL) and differentiation was stimulated by the addition of 100 nmol/mL phorbol 12-myristate 13-acetate (PMA) (Sigma-Aldrich). THP-1 cells were incubated for 48 h at 37°C and 5% CO_2_ prior to infection. M. bovis BCG strains cultured in 7H9 to midlogarithmic phase, were centrifuged and washed with infection medium (RPMI 1640, PAA) containing 1% heat-inactivated FBS. THP-1 cells were infected with M. bovis BCG strains using a multiplicity of infection (MOI) of 10 bacteria per phagocyte at 37°C and 5% CO_2_ in infection medium. Bacteria were slightly centrifuged (2 min, 200 × *g*) onto the cells to initiate a simultaneous contact with phagocytes. Post infection, phagocytes were washed with infection medium and subsequently incubated with Penicillin G (100 units/mL, Sigma-Aldrich) and Gentamicin (0.1 mg/mL, Sigma-Aldrich) for 1 h at 37°C and 5% CO_2_. After washing, the phagocytes were lysed using 0.025% SDS at the indicated time points. The CFU (CFU of released intracellular M. bovis BCG strains was determined by plating the bacteria in appropriate dilutions on 7H10 agar plates.

### Confocal microscopy.

For visualization of free zinc, the THP-1 cells infected with DiI-labeled M. bovis BCG strains were incubated for 1 h with the cell permeant reagent FZ3 (Invitrogen) at a final concentration of 1 mM in PBS. The confocal images were acquired using the Olympus FV1000 confocal microscope. An UPlanSApo100x/1.40 NA oil objective was used to obtain an over sampled 1024 × 1024 image with 49 nm pixel and a *z*-stack with a step size of 120 nm, recorded with Zen software (Carl Zeiss, Inc.), and analyzed with Olympus FV1000 confocal microscope.

### Mouse infection.

WT female SPF C57BL/6 mice were purchased from Chang-sheng Bio (Liaoning, China). The mice weighed 16–18 g and were 6–8 weeks old and housed in a specific pathogen-free facility using standard humane animal husbandry protocols that were approved by the Research Ethics Committee of the College of Veterinary Medicine, Huazhong Agricultural University, Hubei, Wuhan, China (HZAUMO-2019-013). M. bovis BCG strains were cultured until the midlogarithmic phase and were washed three times with PBS containing 0.05% Tween 80. Thirty-six mice were randomly divided into six groups (*n* = 6) and were intratracheally infected with 1 × 10^6^ CFU of WT/pMV261, Δ*ctp*G/pMV261, and OE-*ctp*G strains separately, and data were analyzed as described previously (59).

### Statistical analysis.

Statistical analyses of data were performed using unpaired two-tailed Student's t test with GraphPad Prism 7. Data are expressed in terms of mean ± S.D. Asterisks represent significant difference, *, *P* < 0.05; **, *P* < 0.01; ***, *P* < 0.001; and ns, not significant (*P* ≥ 0.05).

### Data availability.

All data described are presented either within the article or in the supporting information. The transcriptomic data of M. bovis BCG strains under zinc treatment conditions were deposited to the Gene Expression Omnibus (GEO) for the accession No: GSE179966.
