# Supplementary material for: A Novel Zinc Exporter CtpG Enhances Resistance to Zinc Toxicity and Survival in Mycobacterium bovis
Source: Microbiol Spectr. 2022 Apr 4;10(2):e01456-21. doi: 10.1128/spectrum.01456-21 (PMC9045314; doi:10.1128/spectrum.01456-21)
Supplement: SUPPLEMENTAL FILE 1 — Supplemental material. Download SPECTRUM01456-21_Supp_1_seq11.pdf, PDF file, 1 MB [file spectrum01456-21_supp_1_seq11.pdf]

A novel zinc exporter CtpG enhances resistance to zinc toxicity and survival in *Mycobacterium bovis*

Liu Chen[1], Xiaohui Li[1,2], Piao Xu[1], Zheng-Guo He*[,1,2]

[1]College of Life Science and Technology, Huazhong Agricultural University, Wuhan 430070, China

[2]State Key Laboratory for Conservation and Utilization of Subtropical Agro-bioresources, College of Life Science and Technology, Guangxi University, Nanning 530004, China.

*To whom correspondence should be addressed: College of Life Science and Technology, Guangxi University, Nanning 530004, China.

Email: hezhengguo2019@163.com

Tel: +86-771-5639685, Fax: +86-771-3225146

Running title: CtpG contributes to the resistance of zinc toxicity

Keywords: *Mycobacterium bovis*; Zinc; CtpG; Intracellular survive

Supplementary Fig. 1 The genomic and structural information of CtpG in *M. bovis* BCG.

Supplementary Fig. 2 Assays for studying the effects of zinc on *ctpG* expression in *M. smegmatis* and *M. bovis* BCG.

Supplementary Fig. 3 RT-qPCR assays the expression of *ctpG* in *M. bovis* BCG strains.

Supplementary Fig. 4 Construction and characterization of *ctpC*-deleted strain and *ctpG*-deleted strain of *M. bovis* BCG.

Supplementary Fig. 5 The effect of Cd^{2+} in the growth of *M. bovis* BCG strains.

Supplementary Fig. 6 Assays for studying the effect of CtpG on zinc detoxification in *M. bovis* BCG.

Supplementary Fig. 7 Analysis of the effects of zinc on *ctpG* expression in the *cmtR*-deleted strain of *M. bovis* BCG**.**

Supplementary Fig. 8 Assays for the biochemical characterization of CtpG.

Supplementary Fig. 9 Assays for the effect of CtpG on intracellular survival of *M. bovis* BCG in THP-1 macrophages.

Supplementary Fig. 10 Assays for evaluating the effects of CtpG on free Zn^{2+} contents and gene expression in THP-1 macrophages.

Supplementary Fig. 11 Histopathology of lungs of mice infected with *M. bovis* BCG strains.

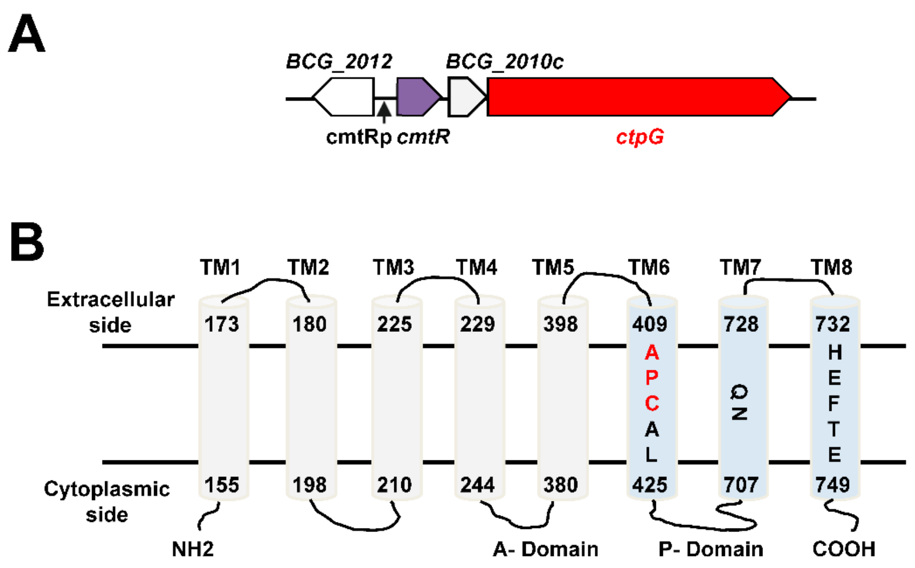

Supplementary Fig. 1 The genomic and structural information of CtpG in *M. bovis* BCG. (**A**) The *cmtR* (*BCG_2011c*) operon genes (*BCG_2009c* - *BCG_2011c*) in *M. bovis* BCG genome was shown. The transcription factor CmtR-regulated region (cmtRp) were indicated by *black* arrows. (**B**) The predicted topology of CtpG shows eight TM helices (1 to 8), locating the N- and C-terminal ends within the cytoplasmic portion. The cytoplasmic domains are represented by stitched lines and the functional motifs of P_{1B}-type ATPase are shown. The amino acids responsible for cation coordination within TM segments 6, 7 and 8 are highlighted in bold.

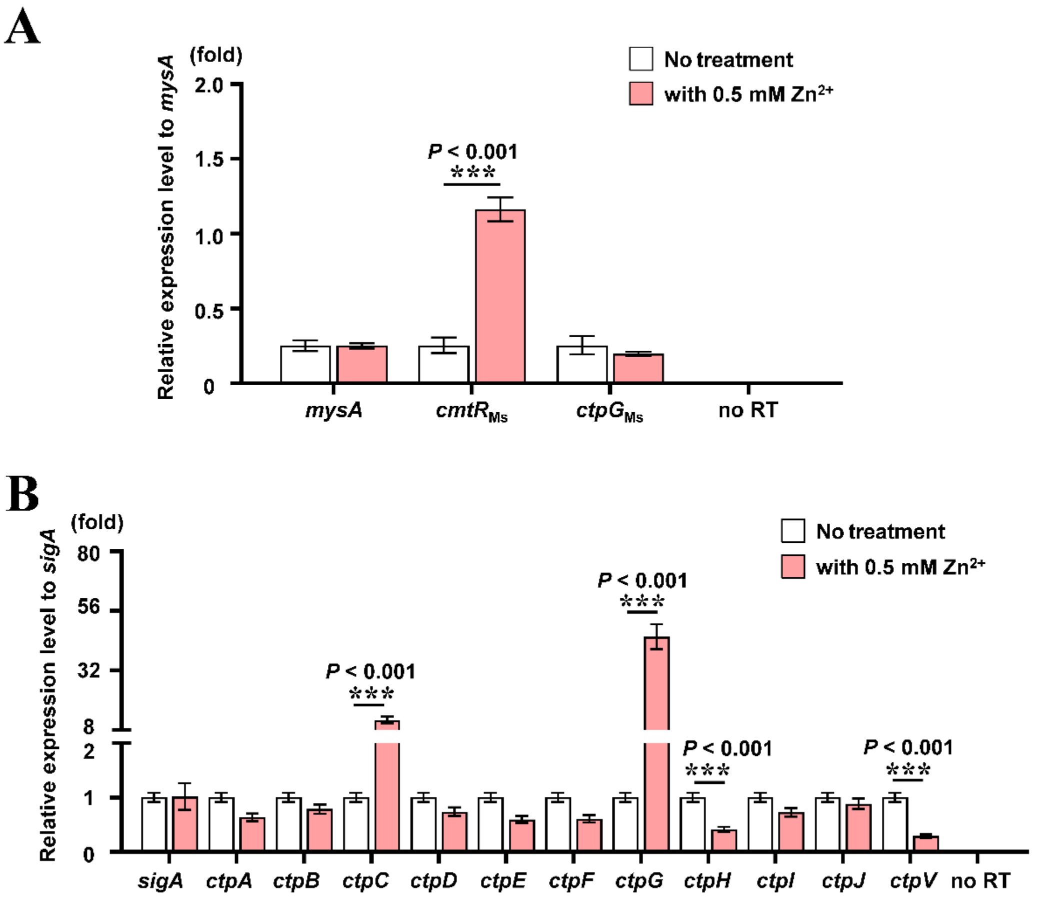

Supplementary Fig. 2 Assays for studying the effects of zinc on *ctpG* expression in *M. smegmatis* and *M. bovis* BCG. (**A**) RT-qPCR assays for studying the effect of zinc on *ctpG* expression in *M. smegmatis*. (**B**) RT-qPCR analysis of *ctp* genes expression in the *M. bovis* BCG upon exposure to zinc. Bacteria were incubated with 0.5 mM $ZnSO_4$ in 7H9 medium for 24 h. Relative expression levels of the genes were normalized by the *sigA* gene, which was used as an invariant transcript control, using the $2^{-\Delta\Delta Ct}$ method. *Error bars* represent the S.D. from three biological experiments. The *P*-values of the data were calculated by unpaired two-tailed Student' s *t* test using GraphPad Prism7. Asterisks denote significant difference (***, $P < 0.001$, two-tailed Student's *t* test) between two groups.

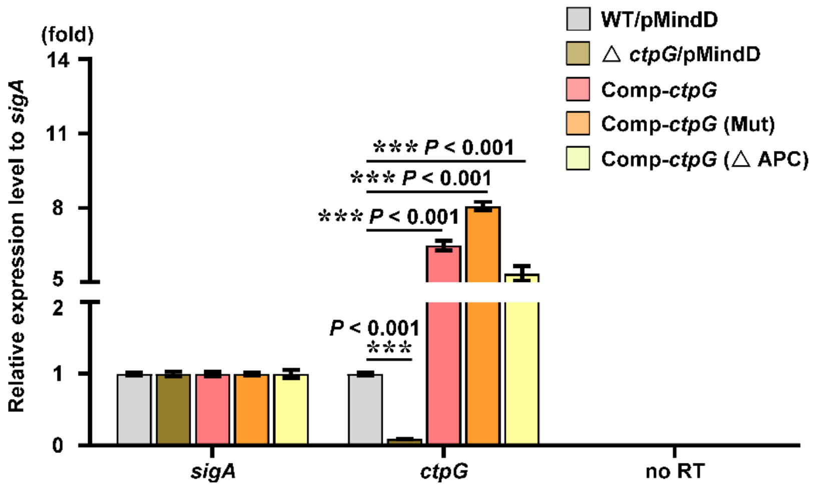

Supplementary Fig. 3 RT-qPCR assays the expression of *ctpG* in *M. bovis* BCG strains. Relative expression levels of the genes were normalized by the *sigA* gene, which was used as an invariant transcript control, using the $2^{-\Delta\Delta Ct}$ method. *Error bars* represent the S.D. from three biological replicates. The *P*-values of the relative expression data were calculated by unpaired two-tailed Student's *t* test using GraphPad Prism 7. Asterisks represent significant difference between two groups: ***, $P < 0.001$.

A

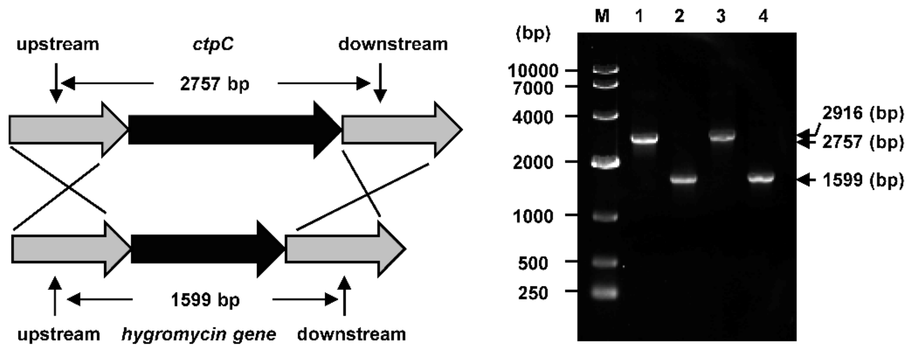

B

>CtpC-UP300-F_DN300-R

AGGGACTGCCGAGGCGACACGACGGTGATCACCGGCTTGGCCGGCGTGACCGCCTACGAGATCTTAAAAAGGCCGCGGC
CAAAGCGCCGCTTCGTCAGACCGCGGTATCGGCAGCAGCGCTGGGTCTGCGCGGAACCCGCAAGGCCGAGGAAGCCGCG
GAATCGGCCCGCCTAAAGGTGGCCGACGTGATGGCCGAGGCTCGTGAGCGCATCGGCGAGGAATCGCCCACTCCAGCGAT
CAGCGACCTGCACGACCACGACCACTGAGCGCCTCGCCACTAGTGCATGCGTGACACAAGAATCCCTGTTACTTCTCGACC
GTATTGATTCGGATGATTCCTACGCGAGCCTGCGGAACGACCAGGAATTCTGGGAGCCGCTGGCCCGCCGAGCCCTGGAG
GAGCTCGGGCTGCCGGTGCCGCCGGTGCTGCGGGTGCCCGGCGAGAGCACCAACCCCGTACTGGTCGGCGAGCCCGGC
CCGGTGATCAAGCTGTTCGGCGAGCACTGGTGCGGTCCGGAGAGCCTCGCGTCGGAGTCGGAGGCGTACGCGGTCCTGG
CGGACGCCCCGGTGCCGGTGCCCCGCCTCCTCGGCCGCGGCGAGCTGCGGCCCGGCACCGGAGCCTGGCCGTGGCCCT
ACCTGGTGATGAGCCGGATGACCGGCACCACCTGGCGGTCCGCGATGGACGGCACGACCGACCGGAACGCGCTGCTCGC
CCTGGCCCGCGAACTCGGCCGGGTGCTCGGCCGGCTGCACAGGGTGCCGCTGACCGGGAACACCGTGCTCACCCCCCAT
TCCGAGGTCTTCCCGGAACTGCTGCGGGAACGCCGCGCGGCGACCGTCGAGGACCACCGCGGGTGGGGCTACCTCTCGC
CCCGGCTGCTGGACCGCCTGGAGGACTGGCTGCCGGACGTGGACACGCTGCTGGCCGGCCGCGAACCCCGGTTCGTCCA
CGGCGACCTGCACGGGACCAACATCTTCGTGGACCTGGCCGCGACCGAGGTCACCGGGATCGTCGACTTCACCGACGTCT
ATGCGGGAGACTCCCGCTACAGCCTGGTGCAACTGCATCTCAACGCCTTCCGGGGCGACCGCGAGATCCTGGCCGCGCTG
CTCGACGGGGCGCAGTGGAAGCGGACCGAGGACTTCGCCCGCGAACTGCTCGCCTTCACCTTCCTGCACGACTTCGAGGT
GTTCGAGGAGACCCCGCTGGATCTCTCCGGCTTCACCGATCCGGAGGAACTGGCGCAGTTCCTCTGGGGGCCGCCAGACA
CCGCCCCCGGCGCCTAAAAGCTTCTAGCAGCCGCAGCCGTGACCACGCCAGGTGCGGATGCCCTGCCAGACCGCGATACC
GGCGATGGCCAGCCCGATCGCGGGGTCAATCCACCAGCCGTTCGACCACACGGCAGTGATCGCCAGCCCAAGCAGAACCG
CGGCGGCCTGAGCAGCACACAGGTAGTTCTGGGTGCCCTCGCCCGCGGTGGCCCCCGATCCCAGCCGTTCACCCACTCGG
TGGTTGGCCCAGCCCAGGACCGGCATCAGCAGCAGTCGAGTCGTAAGCG

>CtpG-UP300-F_DN300-R

AGGACTGACCGTGGTTACGCATGAGCTATTGGTTAAGGCGGCCGGGGCGGTGCTCACCGGACTGGTCGGGGTGAGCGCCT
ACGAAACGCTGCGCAAAGCGCTGGGTACGGCGCCCATTCGCCGGGCGTCGGTGACCGTGATGGAGTGGGGCCTGCGCGG
AACCCGGCGCGCGGAGGCGGCGGCCGAGTCGGCCCGGCTGACAGTCGCCGACGTCGTTGCCGAGGCTCGCGGGCGCAT
CGGTGAGGAGGCGCCCCTGCCCGCTGGCGCCAGGGTCGACGAGTGACGACTGTAGACTAGTGCATGCGTGACACAAGAAT
CCCTGTTACTTCTCGACCGTATTGATTCGGATGATTCCTACGCGAGCCTGCGGAACGACCAGGAATTCTGGGAGCCGCTGG
CCCGCCGAGCCCTGGAGGAGCTCGGGCTGCCGGTGCCGCCGGTGCTGCGGGTGCCCGGCGAGAGCACCAACCCCGTACT
GGTCGGCGAGCCCGGCCCGGTGATCAAGCTGTTCGGCGAGCACTGGTGCGGTCCGGAGAGCCTCGCGTCGGAGTCGGAG
GCGTACGCGGTCCTGGCGGACGCCCCGGTGCCGGTGCCCCGCCTCCTCGGCCGCGGCGAGCTGCGGCCCGGCACCGGA
GCCTGGCCGTGGCCCTACCTGGTGATGAGCCGGATGACCGGCACCACCTGGCGGTCCGCGATGGACGGCACGACCGACC
GGAACGCGCTGCTCGCCCTGGCCCGCGAACTCGGCCGGGTGCTCGGCCGGCTGCACAGGGTGCCGCTGACCGGGAACAC
CGTGCTCACCCCCCATTCCGAGGTCTTCCCGGAACTGCTGCGGGAACGCCGCGCGGCGACCGTCGAGGACCACCGCGGG
TGGGGCTACCTCTCGCCCCGGCTGCTGGACCGCCTGGAGGACTGGCTGCCGGACGTGGACACGCTGCTGGCCGGCCGCG
AACCCCGGTTCGTCCACGGCGACCTGCACGGGACCAACATCTTCGTGGACCTGGCCGCGACCGAGGTCACCGGGATCGTC
GACTTCACCGACGTCTATGCGGGAGACTCCCGCTACAGCCTGGTGCAACTGCATCTCAACGCCTTCCGGGGCGACCGCGA
GATCCTGGCCGCGCTGCTCGACGGGGCGCAGTGGAAGCGGACCGAGGACTTCGCCCGCGAACTGCTCGCCTTCACCTTCC
TGCACGACTTCGAGGTGTTCGAGGAGACCCCGCTGGATCTCTCCGGCTTCACCGATCCGGAGGAACTGGCGCAGTTCCTCT
GGGGGCCGCCAGACACCGCCCCCGGCGCCTAAAAGCTTCGACGCGCGGAATCGTGGAGTGTGTTTGGACCAGCAATAGCG
TCACTGTGACGAAACAGCCGCCGTCTTCTGGAAGTTATACCCGGTTATACTATCTGTATGAAGACAGCTATTTCTCTGCCGGA
TGAGACGTTCGATCGGGTATCGCGGCGTGCGAGTGAGCTCGGCATGAGTCGGTCCGAGTTCTTCACGAAGGCTGCGCAGC
GCTACCTGCACGAGCTGGACGCCCAATGCTCACGTCAGTTAT

Supplementary Fig. 4 Construction and characterization of *ctpC*-deleted strain and *ctpG*-deleted strain of *M. bovis* BCG. **(A)** Validation of the *ctpC*-deleted strain and the *ctpG*-deleted strain by PCR. The wild-type and mutant strains were used as templates to amplify the gene (*ctpC/ctpG*) from -300bp upstream to 300bp downstream by PCR, respectively. Left panel: schematic representation of the recombination strategy for the ctpC deletion from the *M. bovis* BCG genome. Lanes 1

and 3 used wild type *M. bovis* BCG genomic DNA as the PCR template; lanes 2 and 4 used the genomic DNA *of ctpC*-deleted strain and *ctpG*-deleted strain as the PCR template, respectively. (**B**) The sequencing analysis of the PCR products. The PCR products shown in (**A**) (lanes 2 and 4) were purified, then sequenced. The sequenced DNA bases (*ctpC*-UP300-F_DN300-R and *ctpG*-UP300-F_DN300-R) marked in *blue* are the -300 bp upstream or the 300 bp downstream of the deleted gene on the genome, respectively; the hygromycin gene is marked in *red*.

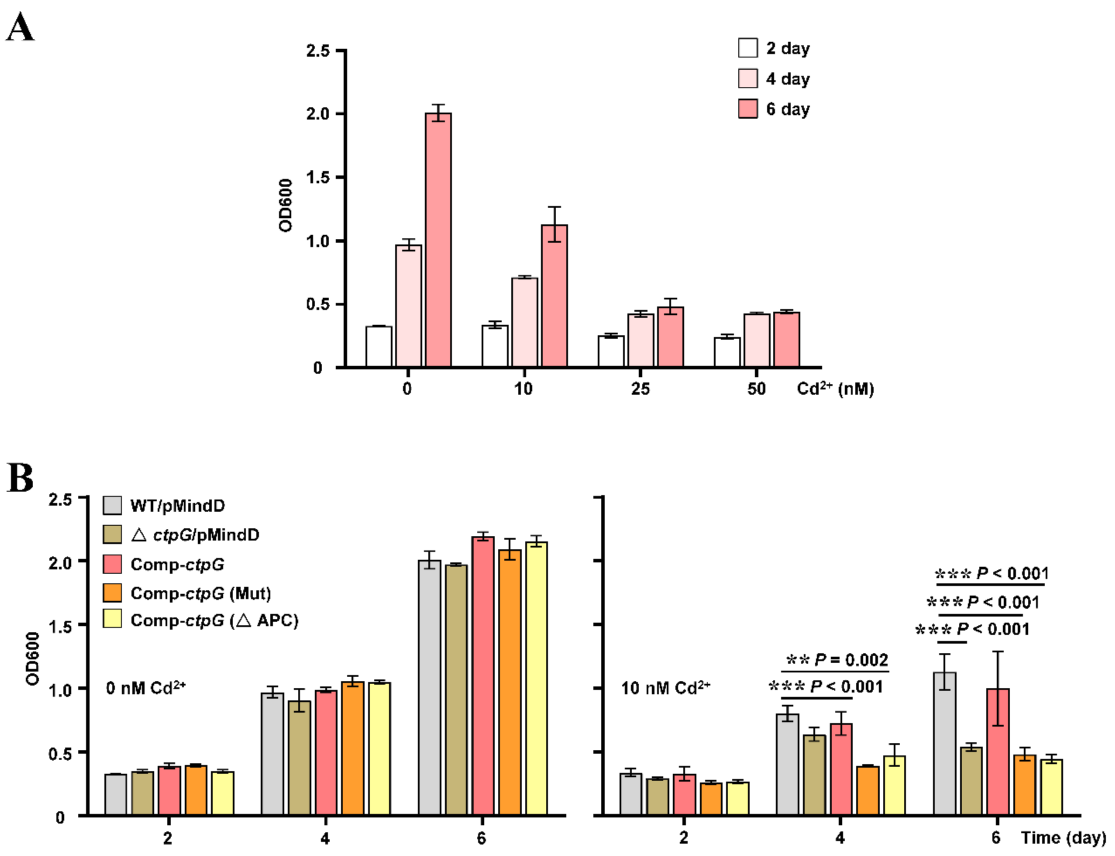

Supplementary Fig. 5 The effect of Cd^{2+} in the growth of *M. bovis* BCG strains. (**A**) Differential sensitivity of *M. bovis* BCG to free Cd^{2+}. *M. bovis* BCG was allowed to grow in 7H9 medium containing 10, 25, 50 nM Cd^{2+}, or without Cd^{2+} supplementation (Control) at various times. (**B**) Assays for studying the effects of *ctpG* deletion on the growth of *M. bovis* BCG strain in 7H9 medium or medium supplemented with 10 nM Cd^{2+} at various times. WT/pMindD represents the BCG/pMindD strain; Δ*ctpG*/pMindD represents the BCG *ctpG*::*hyg*/pMindD strain; comp-*ctpG* represents the BCG *ctpG*::*hyg*/pMindD-*ctpG* strain; comp-*ctpG* (Mut) represents the BCG *ctpG*::*hyg*/pMindD-*ctpG* (Mut) strain, and comp-*ctpG* (ΔAPC) represents the BCG *ctpG*::*hyg*/pMindD-*ctpG* (ΔAPC) strain. *Error bars* represent the S.D. from three biological experiments. The *P*-values of the data were calculated by unpaired two-tailed Student's *t* test using GraphPad Prism7. Asterisks denote significant difference (***, $P < 0.001$, two-tailed Student's *t* test) between two groups.

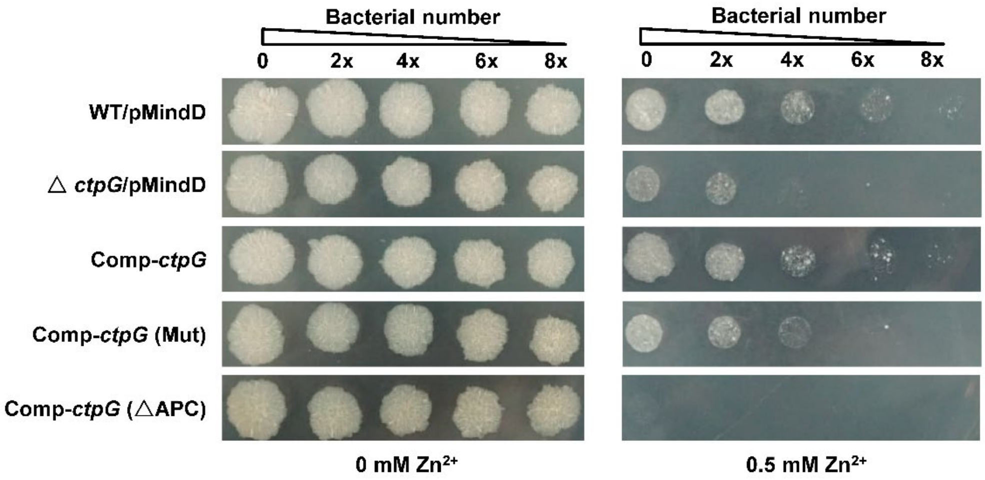

Supplementary Fig. 6 Assays for the effect of CtpG on zinc detoxification in *M. bovis* BCG. Different *M. bovis* BCG strains (mentioned in **Fig. 3**) were serially diluted and spotted on 7H10 plates supplemented with 0.5 mM $ZnSO_4$, or without $ZnSO_4$ supplementation (Control), and cultured at 37 °C for 14 days, then photographed.

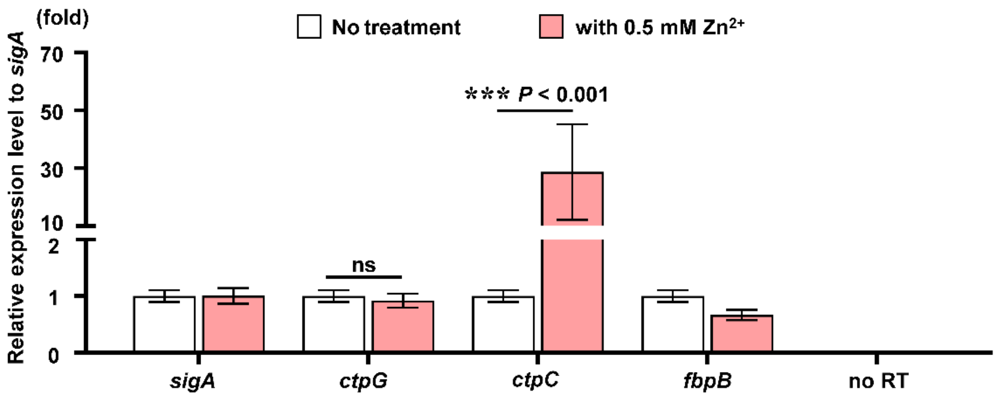

Supplementary Fig. 7 Analysis of the effects of zinc on *ctpG* expression in the *cmtR*-deleted strain of *M. bovis* BCG. RT-qPCR analysis of *ctpG* expression in the *cmtR*-deleted strain of *M. bovis* BCG upon exposure to 0.5 mM $ZnSO_4$. Bacteria were treated as described in **Supplementary Fig. 2A**. *ctpC* and *fbpB* were used as controls. *Error bars* represent the S.D. from three biological experiments. The *P*-values of the data were calculated by unpaired two-tailed Student' s *t* test using GraphPad Prism7. Asterisks denote significant difference (***, $P < 0.001$, two-tailed Student' s *t* test) between two groups.

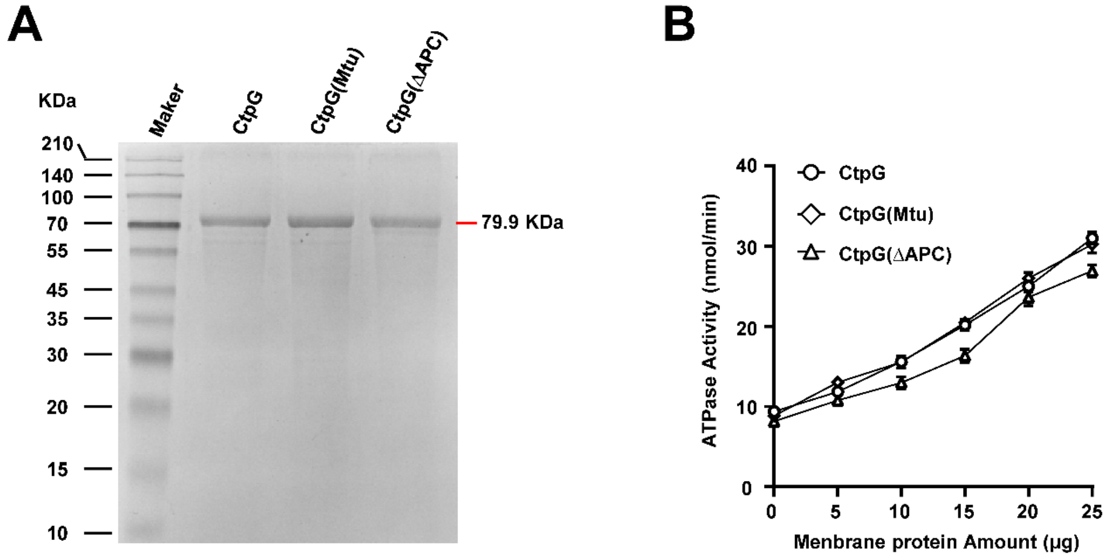

Supplementary Fig. 8 Assays for the biochemical characterization of CtpG. (**A**) CtpG, CtpG (Mut), and CtpG (Δ APC) purification. 10 μg of protein was resolved in SDS-PAGE and Coomassie Brilliant Blue (CBB)-stained. (**B**) The enzymatic parameters of CtpG, CtpG (Mut), and CtpG (Δ APC) were evaluated with amount of the membrane protein, respectively.

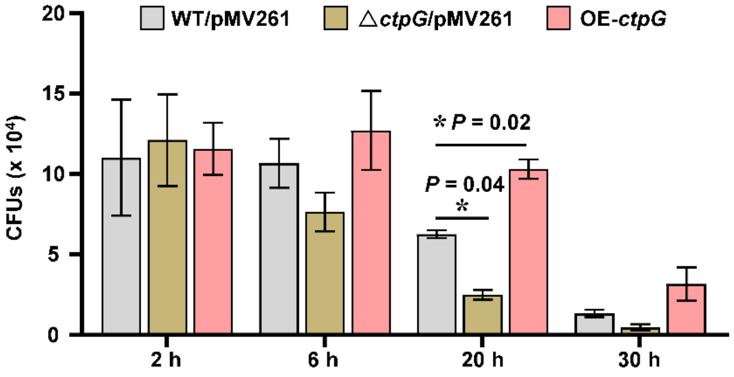

Supplementary Fig. 9 Assays for the effect of CtpG on intracellular survival of *M. bovis* BCG in THP-1 macrophages. The intracellular survival assays were performed as described in **Fig. 7A**. WT/pMV261 represents the BCG/pMV261 strain; Δ*ctpG*/pMV261 represents the BCG *ctpG*::*hyg*/pMV261 strain; OE-*ctpG* represents the BCG/pMV261-*ctpG* strain. The *P*-values of the data were calculated by unpaired two-tailed Student' s *t* test using GraphPad Prism7. Asterisks denote significant difference (*, $P < 0.05$, two-tailed Student' s *t* test) between two groups.

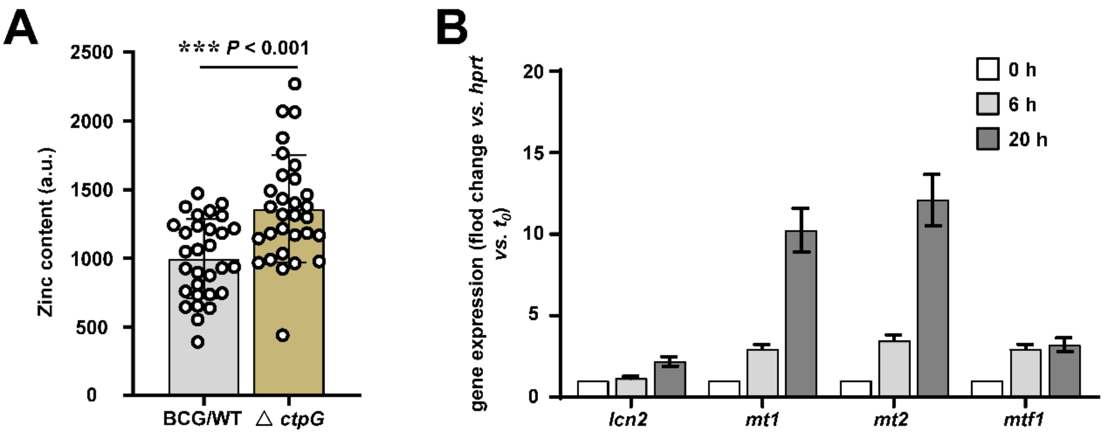

Supplementary Fig. 10 Assays for evaluating the effects of CtpG on free Zn^{2+} contents and gene expression in THP-1 macrophages. (**A**) FZ3 signal quantification (in arbitrary units) from ~ 35 blind-scored THP-1 macrophages from three random fields. (**B**) Quantitative PCR analysis of *lcn2* mRNA, *mt1* mRNA, *mt2* mRNA and *mtf1* mRNA in THP-1 macrophages infected with *M. bovis* BCG at the indicated post-infection time points. The data shown are means S.D. of the signal measured from 35 cells and were analyzed with Student's *t* test using GraphPad Prism7. Asterisks denote significant difference (***, $P < 0.001$, two-tailed Student' s *t* test) between two groups.

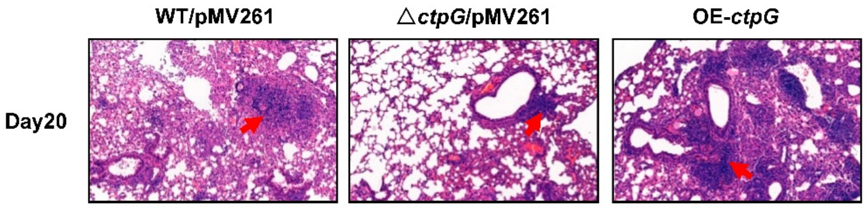

Supplementary Fig. 11 Histopathology of lungs of mice infected with *M. bovis* BCG strains. Micrographs showed the representative images obtained from *M. bovis* BCG strains-infected mice (mentioned in **Fig. 8B**) for 20 days. Arrows indicate the foci of cellular infiltration.
